# Application of the Asymmetric Pictet–Spengler Reaction in the Total Synthesis of Natural Products and Relevant Biologically Active Compounds

**DOI:** 10.3390/molecules23040943

**Published:** 2018-04-18

**Authors:** Majid M. Heravi, Vahideh Zadsirjan, Masumeh Malmir

**Affiliations:** Department of Chemistry, Alzahra University, Vanak, Tehran 1993893973, Iran; masi.malmir@yahoo.com

**Keywords:** tetrahydroisoquinolines, asymmetric Pictet–Spengler reaction, total synthesis, natural products, biologically active compounds, β-aryl ethylamine

## Abstract

Tetrahydroisoquinolines are the framework of numerous natural products predominantly alkaloids, an important and one of the most wide spread families of naturally occurring compounds in the plant kingdom. Tetrahydroisoquinolines are commonly constructed through an old reaction, the so-called Pictet–Spengler Reaction (PSR). In this reaction, a β-aryl ethylamine undergoes an acid mediated condensation with a suitable aldehyde or ketone, followed by ring closure. In this review, we aim to highlight the applications of the asymmetric variant of this old name reaction in the total synthesis of natural products, chiefly, alkaloids, which exhibit significant biological properties.

## 1. Introduction

Indole and isoquinoline derivatives, historically and currently are synthesized *via* an old reaction, the so-called Pictet–Spengler reaction (PSR). This old reaction nowadays has found a few new perspectives. It is currently and frequently used as a vital step in the total synthesis of natural products especially in those bearing indole and isoquinoline alkaloids as scaffolds in their complex structures [1,2,3].

**Scheme 1:**
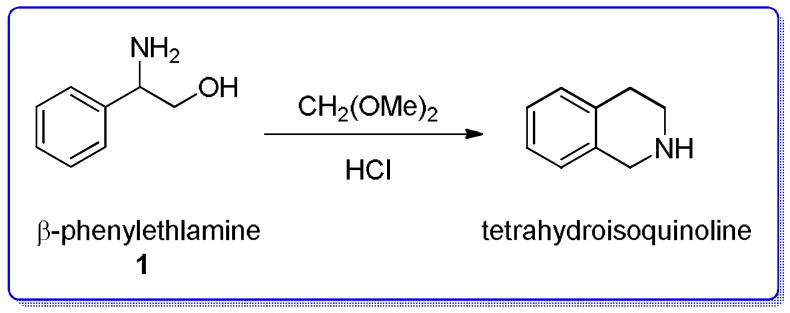
The first example of the Pictet–Spengler Reaction (PSR), resulted in the synthesis of the tetrahydroisoquinoline (THIQ).

The PSR was discovered more than a century ago, in 1911 by Ame Pictet and Theodor Spengler [4]. As illustrated in Scheme 1, they condensed phenethylamine **1** with methylal that is CH_2_(OMe)_2_ to obtain tetrahydroisoquinoline **2** [4]. Commonly, the PSR is a chemical reaction in which a β-arylethylamine such as tryptamine is cyclocondensed with an aldehyde or ketone under acidic and thermal conditions [5,6]. Some reactive substrates give acceptable yields even at physiological conditions [7]. Remarkably, the PSR can be considered as a special type of Mannich reaction.

The PSR has been initially used for the synthesis of tetrahydroisoquinolines in the total synthesis of indole and isoquinoline alkaloids [3]. Since it was disclosed that isoquinoline alkaloids are generated in plants biosynthetically through the condensation of β-arylethyl amines with carbonyl compounds, this reaction has found useful practicality in the total synthesis of naturally occurring compounds bearing isoquinolines in their complex structures [8]. Typically, tryptamine and secologan are condensed to generate strictosidine, stereoselectively. As a matter of fact, tryptamine is the common precursor for the syntheses of all indole alkaloids [9,10].

The asymmetric type of this reaction was applied in various instances for stereospecific acceptance of this method and has played a key role in the total synthesis of various indole, oxindole, bisindole and in general, alkaloids [11,12,13,14,15,16,17,18]. In 2016, Dalpozzo reported an overview of the asymmetric PSR, in which the chirality was induced by optically pure amines or carbonyl compounds, obtained from natural sources or from asymmetric synthesis to assemble the reaction partners [19].

Alkaloids, are undoubtedly, the most important and widespread family of natural products in the kingdom of plants. A plethora of alkaloids has been extracted, isolated, screened and their biological properties have been evaluated, to mostly exhibit remarkable and various biological and pharmacological activities, thus, they have exceptionally attracted the attention of organic synthetic and bio-organic chemists. Alkaloids are natural products which include mostly one or more basic nitrogen atoms [20]. Moreover, this family includes some relevant neutral compounds and some of them can be even weakly acidic [21]. Alkaloids, in addition to carbon, hydrogen and nitrogen, may contain other elements such as oxygen, sulfur and even infrequently may have unusual elements such as, chlorine, bromine, and phosphorus.

Alkaloids are provided by a vast range of organisms including bacteria, fungi, plants and animals. They are typically purified from crude extracts of these organisms through acid–base extraction. Alkaloids exhibit a wide variety of biological activities for example quinine, the known anti-malaria agent, anticancer e.g. homoharringtonine [22], cholino mimetic such as galantamine [23], vasodilatory like vincamine, antiarrhythmic such as quinidine, analgesic such as the notorious morphine [24] antibacterial such as. chelerythrine [25], and antihyperglycemic like piperine [26]. Many of them have found found applications in traditional or modern medicinal chemistry or as starting points in novel drug discovery. Many others show also psychotropic (e.g. psilocin) and stimulant activities for example the notorious cocaine and morphine, the familiar caffeine, and the carcinogenic nicotine. Several of them are also used in entheogenic rituals or as medicines. Several of the alkaloids have been screened and evaluated as being toxic for instance atropine and tubocurarine [27]. Importantly, alkaloids are uniquely included in metabolic systems in humans and other animals. Most alkaloids have a bitter and unpleasant taste [28].

Among the accumulation of alkaloids, isoquinoline and β-carboline alkaloids have attracted much interest because of their extensive existence in plants and even in the animal protectorate. Furthermore, the above-mentioned alkaloids and particularly those including the isoquinoline scaffold commonly exhibit a comparative dominance of physiological properties [29]. Their biological activities, which range distinctly from extremely toxic, for example strychnine [30] to antihypertensive ajmalicine [31] and reserpine [32]. Moreover alkaloids show cytotoxic properties exhibited by vincoleucoblastine and vincristine are components applied in the cancer chemotherapy protocol [33,34]. Strikingly and fascinatingly, all these biologically active alkaloids are principally generated indoles from tryptamine attained from tryptophan and a terpenoid part that in turn is biosynthesized *via* the iridoid glucoside secologanin. Tryptamine and secologanin are condensed stereoselectively to create strictosidine, which is used as precursor for the total synthesis of virtually all alkaloids bearing the indole moiety in their structures [10,35].

The total synthesis of natural products including multiple generations of chiral centers remains attractive in both industrial research and development (R & D) as well as academic research [36,37,38]. In this line, predominantly, multi-component cascade, tandam, domino and sequential reactions, in the total synthesis of natural products and synthetic scaffolds within complex molecules, are involved [39,40,41,42]. Alkaloids constituting the indole moiety are a significant class of natural products. Particularly, the indolo[2,3-*a*]quinolizidine scaffold is a common frame work present in numerous biologically active significant products [43,44].

We are particularly interested in heterocyclic chemistry [45,46,47,48,49,50,51] and heterocyclic compounds exhibiting comparatively acceptable biological properties [51,52,53]. Recently, we have underscored the applications of several name reactions and stereoselective synthesis in the total synthesis of biologically active naturally occurring compounds [54,55,56,57,58,59]. In this report, we try to underline the most recent and current applications of another old but significant name reaction, PSR as a vital step in the total synthesis of biologically active natural products, specially, alkaloids.

## 2. Pictet–Spengler Reaction in the Total Synthesis of Natural Products 

### 2.1. Indole Scaffold

Several alkaloids contain indole as a scaffold in their structure. In addition, numerous indole alkaloids involve isoprene groups and are therefore called terpene indole or secologanin tryptamine alkaloids. Notably, more than 4100 various alkaloids have been recognized thus, it is one of the largest groups in the plant kingdom [60]. Several of them contain important physiological properties and some of them have been applied in medicine. Remarkably, the amino acid tryptophan is the biochemical precursor of indole alkaloids [61].

(+)-Ajmaline **7** was extracted in 1931 from the roots of *Rauwolfia serpentine* [62] and was found to contain six rings and four heteroatoms, along with stereogenic centers. Significantly [62,63], it is a clinically important cardiovascular indole alkaloid [64,65,66] having historical prominence and is correlated to the sarpagine bases [67]. The most significant action of ajmaline is an anti-arrhythmic influence on the heart which is just less marked than that of the prescribed drug propranolol [66].

**Scheme 2:**
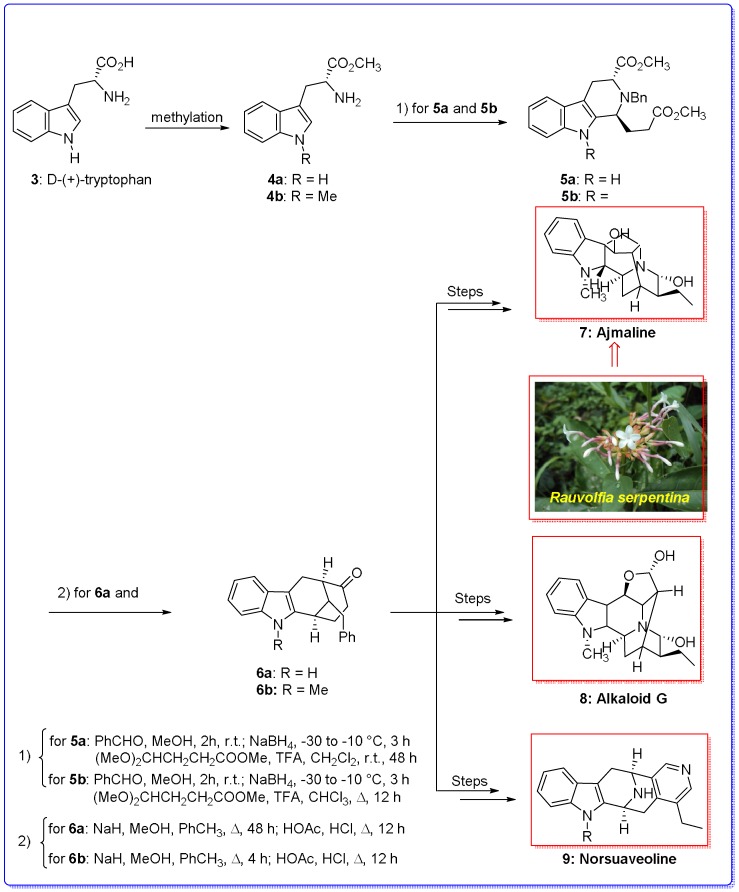
Total synthesis of ajmaline **7**, alkaloid G **8** and norsuaveoline **9**.

Furthermore, alkaloid G **8** was extracted from plant cell cultures of *Rauwolfia serpentina* Benth by Stöckigt and co-workers [68], which upon feeding tests with ajmaline is also structurally analogous to **7**. Cook and co-workers in 1999 reported a common method (oxyanion-Cope strategy) for the formation of ajmaline indole alkaloid [69]. (+)-Ajmaline **7** and alkaloid G **8** as well as norsuaveoline **9** were provided from d-(+)-tryptophan in an enantiospecific approach through the enantioselective PSR and a stereo controlled oxyanion-Cope rearrangement as the main steps. With this route, total synthesis of natural products ajmaline **7**, alkaloid G **8** and **9** were achieved starting from d-(+)-tryptophan **3**. Then, d-(+)-tryptophan **3** was converted into the tryptophan methylester **4b** that was transformed to the *N*_b_-benzyltryptophan derivative on stirring tryptophan methylester **4b** with benzaldehyde in methanol at ambient temperature, followed by reduction of the imine provided by using sodium borohydride as reductive agent. Next, HOAc was added to the reaction mixture to destroy any remaining NaBH_4_ and the solvent was evaporated under reduced pressure. In the following, methyl 4,4-dimethoxybutyrate, chloroform, and trifluoro acetic acid were added directly to the reaction vessel and the solution was refluxed to produce the *trans* diester **5b** in excellent yield (overall yield >85%). The *trans* isomer **5b** *via* epimerization and a Dieckmann cyclization produced the β-ketoester after that the solvent was removed under reduced pressure. Noticeably, HOAc and HCl were added cautiously to the reaction vessel and, on heating, ketone **6b** was formed in more than 98% enantioselectivity (overall yield from **4** > 74%). The ketone **6a** was also synthesized by a similar method. Significantly, five chemical conversions from tryptophan methylester **4** to the (−)-tetracyclic ketone **6** were performed in two reaction vessels. The usefulness of this enantiospecific two-pot sequence through the *trans* transfer of chirality in the enantioselective PSR is the key feature because these reactions can be accomplished on a multi-hundred gram-scale to give the (−)-tetracyclic ketone **6a** or **6b**, that can be used as an easily accessible initiating precursor for the construction of optically pure sarpagine/macroline/ajmaline alkaloids. Moreover, d-(+)-tryptophan and l-(−)-tryptophan are easily accessible from marketable sources allowing entry into both antipodes of the natural products for biological examination. Lastly, (+)-ajmaline **7** and alkaloid G **8** were synthesized in 93% and 92% yield, respectively. In addition, the total synthesis of norsuaeoline **9** was accomplished in 10 reaction vessels with an overall yield of 28%. Remarkably, for the formation of these indole alkaloids a stereospecific PSR/Dieckmann method was used to make the main intermediate, (−)-N_b_-benzyl tetracyclic ketone **6a** or **6b**, which was transformed to (+)-ajmaline **7**, alkaloid G **8** and norsuaveoline **9** (Scheme 2) [69].

During the last decades, numerous macroline/sarpagine correlated indole alkaloids were isolated from different species of *Alstonia* [67,70,71]. Interestingly, the macroline/sarpagine alkaloids originated as a result of folktales, demonstrating the medicinal activities of the plants from which these alkaloids were extracted [72,73]. A range of alkaloids from *Alstonia angustifolia* were demonstrated to contain antiprotozoal property against *Plasmodium falciparum* or *Entamoeba histolytica* in vitro [74], whereas other sarpagine alkaloids were known to include sedative, ganglionic blocking, antibacterial or hypoglycemic properties [70]. These alkaloids were examined for activity against HIV [75,76,77] and cancer [75]. Noticeably, talpinine **15** is a characteristic illustrative of a seven-membered sub group of talpinine-related alkaloids that show pharmacological properties [70]. Inappropriately, to date, none of these bases have been provided or examined in vivo in detail. Talcarpine **16** is also a related macroline/sarpagine indole alkaloid whose total synthesis has not been reported so far [67,71]. Furthermore, two closely related ring-A methoxylated derivatives of alstonerine [71] contain parts of the bisindoles macralstonine and alkaloid H [67]. Macralstonine and its derivatives, in fact, were known to show significant hypotensive and antiamoebic properties, respectively [78,79,80]. The correlated monomer anhydro macrosalhine-methine **18** results in them being part of the bisindole (−)-macrocarpamine.

The asymmetric total synthesis of talpinine **15** and talcarpine **16** was performed in 13 steps (11 reaction vessels) in 10% and 9.5% overall yields, respectively. Furthermore, this synthetic method was used for the synthesis of alstonerine **17** and anhydromacrosalhine methane **18** in 14 reaction steps (12% overall yield) and 12 steps (14% overall yield), respectively. d-(+)-Tryptophan **3** acted here both as the chiral auxiliary and the initiating precursor that provided a simple pathway [from l-(−)-tryptophan] to the antipodes of these alkaloids, if desired. The stereo specific transformation of **3** into **14a/b** on a multi-hundred gram-scale occurred in only two reaction vessels.

**Scheme 3:**
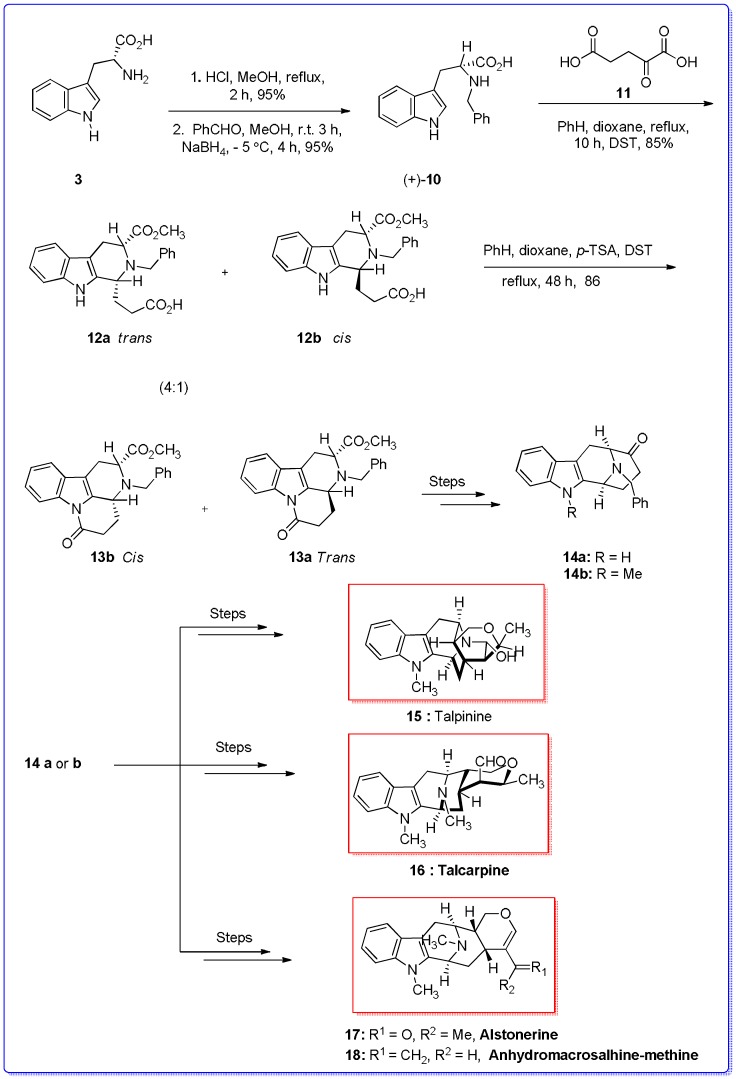
Total synthesis of natural products talpinine **15**, talcarpine **16**, alstonerine **17**, and anhydro macrosalhine methine **18.**

Total synthesis of talpinine **15** was initiated from d-(+)-tryptophan **3**. After several steps, compound **10** was formed. To introduce the corresponding stereocenter at the C1 position having similar chirality to that of the natural macroline/sarpagine/ajmaline alkaloids, Soerens and co-workers [81] and Sakai and co- workers [82] performed the Pictet–Spengler condensation between N_a_-H, N_b_-benzyl tryptophan methylester **10** and α-ketoglutaric acid **11**. This approach gave a mixture of ester acids (*trans*-**12a**/*cis*-**12b**) and δ-lactams (*trans*-**13a**/*cis*-**13b**) with satisfactory *trans* diastereoselectivity (Scheme 3) [83].

The sarpagine alkaloid (+)-vellosimine **21** was extracted from the tree *Geissospermum Vellosii* in 1958 by Rapoport and co-workers [84,85]. Amorphous extracts of this bark, found as *paopereira*, have long enjoyed a reputation as a febrifuge [86]. Also, in Brazilian folk medicine it was described to have curare-like properties [87]. Moreover, (+)-vellosimine was extracted from different species of *Rauwolfia* that are widely dispersed thought Asia and Africa [88,89,90,91]. These plants are broadly used in traditional Chinese medicine for the treatment of hypertension [89,92] neuralgia, and migraine [93].

The first stereospecific total synthesis of the *N_a_*-H functionalized indole alkaloid (+)-vellosimine **21** was performed by Cook and co-workers in 2000 from market purchasable d-(+)-tryptophan methylester **19** in seven reaction steps in 27% overall yield through the enantioselective PSR and a stereo controlled intramolecular Pd (enolate-catalyzed) coupling reaction as main steps [94].

**Scheme 4:**
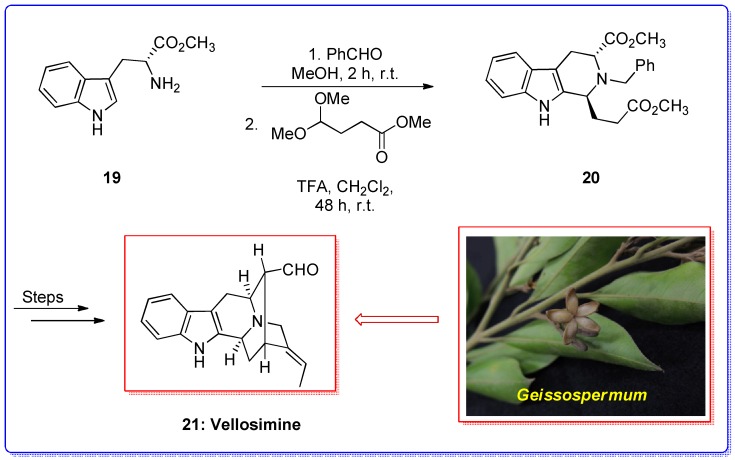
Total synthesis of vellosimine **21.**

Significantly, the chirality at C-3 and C-5 was developed by the enantioselective PSR and Dieckmann reaction in a two-pot approach [95]. Next, benzylation of the *N*_b_-amino scaffold of d-tryptophan methyl ester **19** afforded *N*_a_-H,*N*_b_-benzyl d-tryptophanmethyl ester, that was readily transformed to the *trans* diastereomer **20** with 100% diastereoselectivity based on the improved conditions of the PSR (83% yield). Upon several steps, the *trans* diester afforded the natural product vellosimine **21** (Scheme 4) [94].

**Scheme 5:**
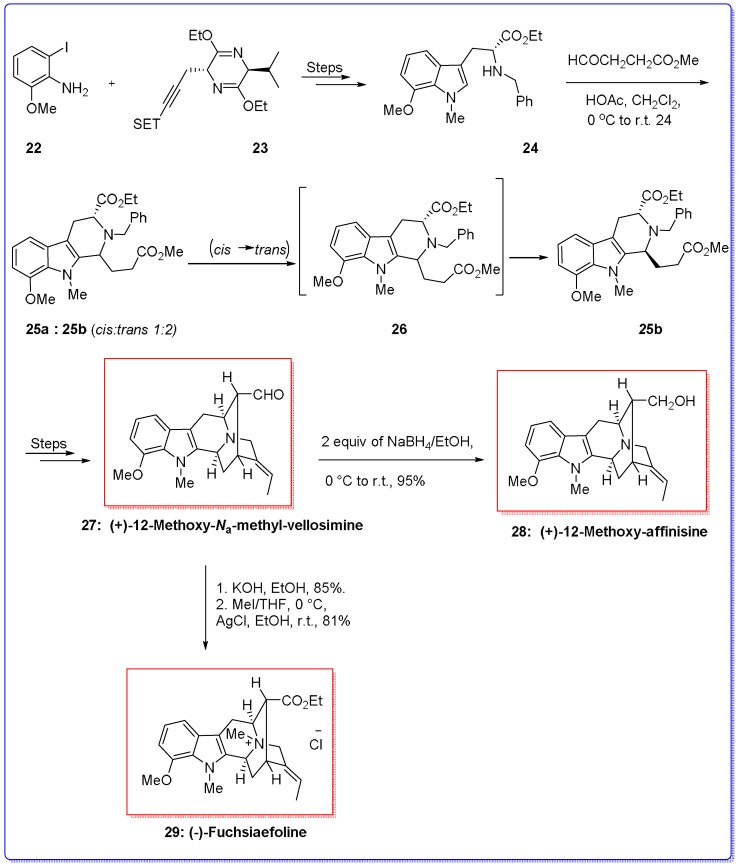
Total synthesis of natural products (+)-12-methoxy-*N*_a_-methyl-vellosimine **27**, (+)-12-methoxy-affinisine **28** and (−)-fuchsiaefoline **29.**

The asymmetric synthesis of 7-methoxy-d-tryptophan was performed using a combination of the Larock hetero annulations method with a Schöllkopf-relied chiral auxiliary in satisfactory yield. Remarkably, this ester was used in the first total synthesis of (+)-12-methoxyaffinisine, (+)-12-methoxy-*N*_a_-methyl vellosimine, and (−)-fuchsiaefoline in a regiospecific, stereospecific approach in high overall yield. The enantioselective PSR and enolate-driven Pd-mediated cross coupling methods acted as the main steps. In this route, initially, 2-iodo-6-methoxyaniline **22 [96]** and the propargyl-functionalized Schöllkopf chiral auxiliary **23 [97]** reacted to provide *N*_b_-benzylester **24**, after several steps [98]. In the following, the Pictet–Spengler condensation of aldehyde and the *N*_b_-benzylamine **24** occurred using HOAc in dichloromethane to provide a mixture (at C-1) of *cis*-**26a** and *trans*-**26b** diesters in approximately quantitative yield in a ratio of 1:2. Once trifluoro acetic acid/dichloromethane was used in this stage instead of acetic acid/dichloromethane, decomposition of a considerable amount of the 7-methoxytryptophan **24** was detected. Mechanistic studies on the carbocation-catalyzed *cis*/*trans* isomerization [99,100], showed that once the PSR was completed, five equivalents of trifluoroacetic acid had to be added to the reaction mixture to epimerize the *cis* diastereomer **26a** into the corresponding *trans* diastereomer **26b**. Finally, the formation of (+)-12-methoxy-*N*_a_-methyl-vellosimine **27**, (+)-12-methoxy-affinisine **28**, and (−)-fuchsiaefoline **29** was performed (from D-tryptophan) in 7,8, and 9 reaction steps, respectively. The enantioselective PSR and an enolate-driven Pd-catalyzed cross-coupling reaction are the two essential stages used to develop the correct stereochemistry in the senatorially occurring compounds (Scheme 5) [98].

A range of bisindole alkaloids extracted from *Alstonia* [70,101] species were exhibited to show anti-malarial properties [74] against a drug resistant (K-1) strain of *Plasmodia falciparum* comprising macrocarpamine, villalstonine, and macralstonine *O*-methylether [102,103]. Bisindoles have noteworthy importance since they show more significant biological properties than the monomeric parts that contain them [74,102,103], which is reminiscent of the significant anti-tumor properties of the *Vinca* alkaloids, vinblastine, and vincristine. The bio mimetic coupling reaction of macroline with the obligatory monomeric alkaloid to give the *Alstonia* bisindoles villalstonine [104,105] macralstonine [106] and alstonisidine [104,107] was initiated by LeQuesne. These alkaloids were synthesized from the distinctive attack of a monomeric part at C(9) of the sarpagine **33a-c** ring system. The latter ring-A oxygenated indole derivatives are not that stable, therefore only trace amounts of the bisindoles (for example **35**) [107,108] have been provided from plants.

The asymmetric stereospecific total synthesis of majvinine **33a**, 10-methoxyaffinisine **33b**, and *N_a_*-methylsarpagine **33c** were developed by Cook and co-workers in 2002 [109]. In addition, this strategy led to the total synthesis of the *Alstonia* bisindole macralstonidine **35**.

This approach is the initial stereospecific synthesis of ring-A alkoxylated indole alkaloids in the sarpagine series. This methodology is distinctive because the natural series (**33a**, **33b**, **33c**) can be synthesized from the inexpensive l-valine and the stereochemistry of the E-ethylidene function can be literally controlled by the enolate catalyzed Pd^0^ cross coupling route. The method to synthesize the bisindole **35** is doubly convergent because the similar key tereochemical methods (enantioselective PSR and enolate catalyzed Pd^0^ method) were used [110] to provide both monomeric parts that could be coupled through the pioneering work of LeQuesne and Garnick [107].

The synthesis started with the easily accessible 3-methyl-5-methoxy indole, which is synthesized [111] through the Japp Kingemann/Fischer indole method [112]. After several steps, *N*_a_-methyl-5-methoxy-d-tryptophanethylester **31** was formed. Favorably, the conversion of **31** into the corresponding *trans* diester **32** should have followed the well-documented trans transfer of chirality in the enantioselective PSR [99] in a direct technique; though, this was not the case. The presence of the 5-methoxy group in **31** assisted the PSR (with benzaldehyde); furthermore, this 5-methoxy indole system was not stable in trifluoro acetic acid/dichloromethane for prolonged periods of time. Noticeably, if amine **31** was transformed to the desired *N*_b_-benzylimine with benzaldehyde/ethanol at ambient temperature [99], important quantities of the 1-phenyltetrahydro β-carbolines were provided [110]. Although, if the imine was provided at 0 °C, followed by reduction at −5 °C, merely the desired *N*_b_-benzyl analogue was detected. Next, compound **31** was not stable in dichloromethane/trifluoro acetic acid for prolonged periods, and the PSR occurred with the aldehyde (instead of the acetal [99]) in acetic acid/dichloromethane. When the cyclization was accomplished to give a mixture of *trans* and *cis* diastereomers, a few drops of trifluoro acetic acid were added [99,110] to assist epimerization (at C-1)of the *cis* isomer into the corresponding *trans* diastereomer (>98% *de*).

**Scheme 6:**
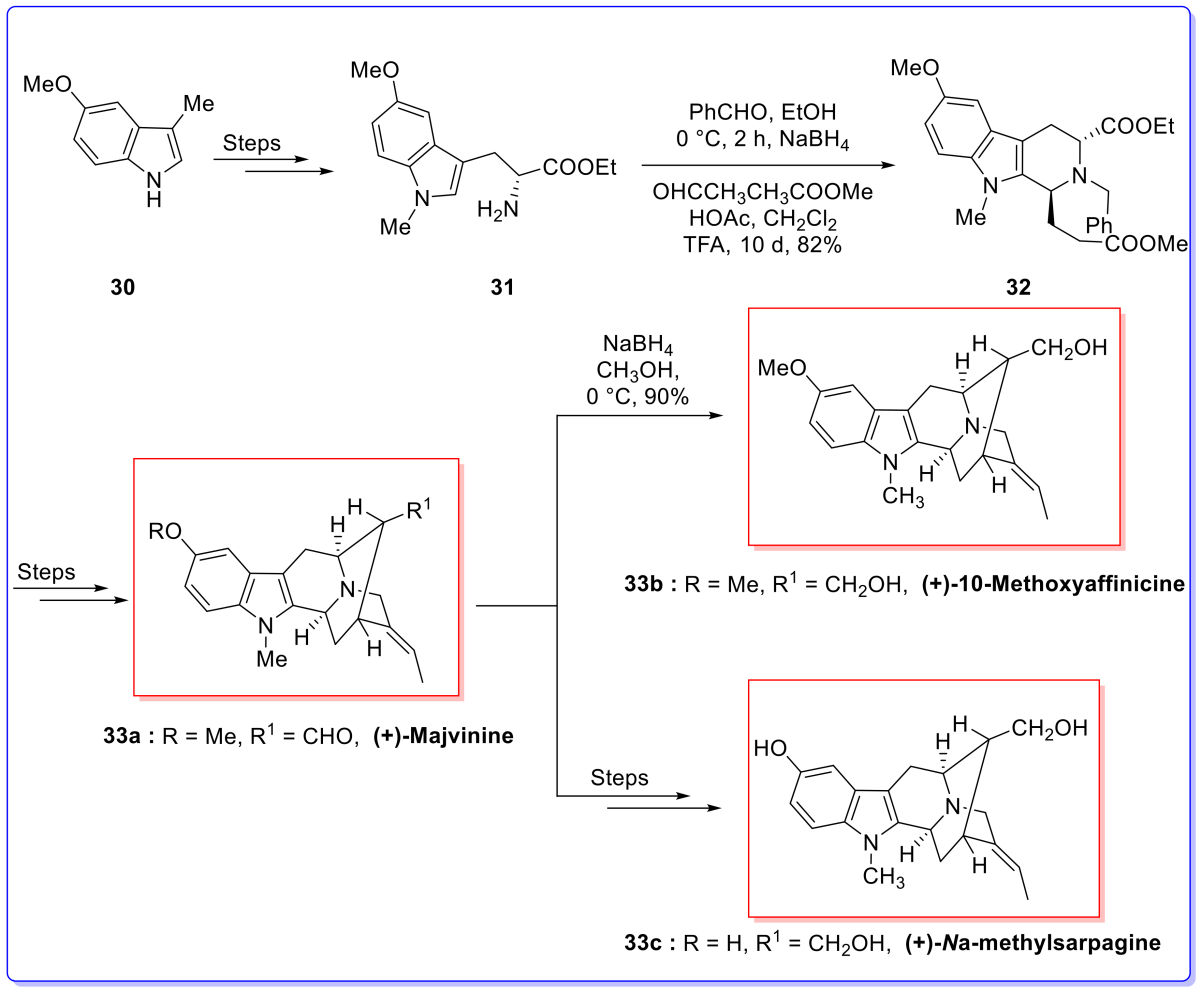
Total synthesis of natural products (+)-majvinine **33a**, (+)-10-methoxyaffinicine, and (+)-*N*_a_-methylsarpagine **33c**.

After several reaction sequence involving the Wittig/hydrolysis/epimerization reaction, (+)-majvinine **33a** was obtained. (+)-Majvinine **33a** (obtained from tryptophanethyl ester **31**) was synthesized in gram quantities (8 steps) in 28% overall yield and acted as the key, stable intermediate for the formation of other naturally occurring compounds in this sequence (Scheme 6) [109].

**Scheme 7:**
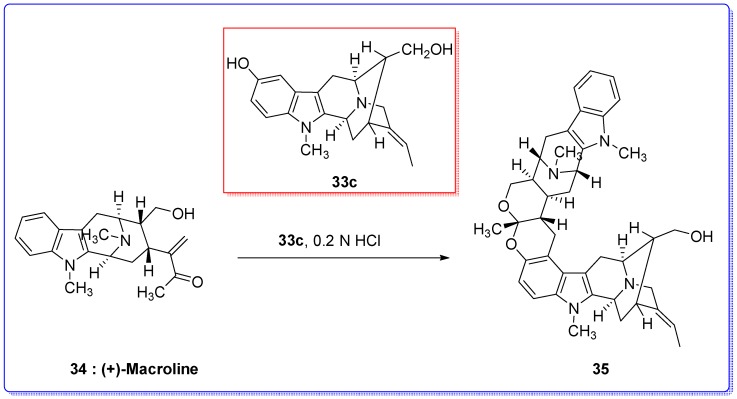
Total synthesis of bisindole macralstonidine **35** using PSR as the key step.

Besides, the alkaloid (+)-majvinine **33a** has been used for the total synthesis of (+)-*N*_a_-methyl sarpagine **33c**. Remarkably, (+)-macroline can be reacted with *N*_a_-methylsarpagine **33c** based on the bio mimetic conditions of Garnick [107] to give **35**; therefore, this provides the initial total synthesis of a bisindole alkaloid in the *Alstonia* groups. More significantly, the *trans* transfer of chirality using the Schöllkopf chiral auxiliary once coupled with the *trans* transfer that occurs in the enantioselective PSR necessarily defines that natural ring-A alkoxylated indoles can be synthesized from inexpensive l-valine whereas d-valine is vital for the antipodal series (Scheme 7) [109].

Strychnofoline belongs to a family of naturally occurring compounds, extracted from the leaves of *Strychnos usambarensis* [113], that shows antimitotic property against cultures of mouse melanoma and Ehrlich tumor cells with strychnofoline exhibiting the uppermost properties [114]. An important structural aspect of these and correlated spirotryprostatin alkaloids [115] is the existence of the aspiro[pyrrolidin-3,3′-oxindole] core.

Carreira and co-workers in 2002 demonstrated a significant synthesis of the antitumor alkaloid (±)-strychnofoline. A key feature of the development of the extremely convergent method described, is the coupling reaction of acyclicimine with spiro[cyclopropan-1,3′-oxindole], which occurs in a highly diastereoselective manner [116].

**Scheme 8:**
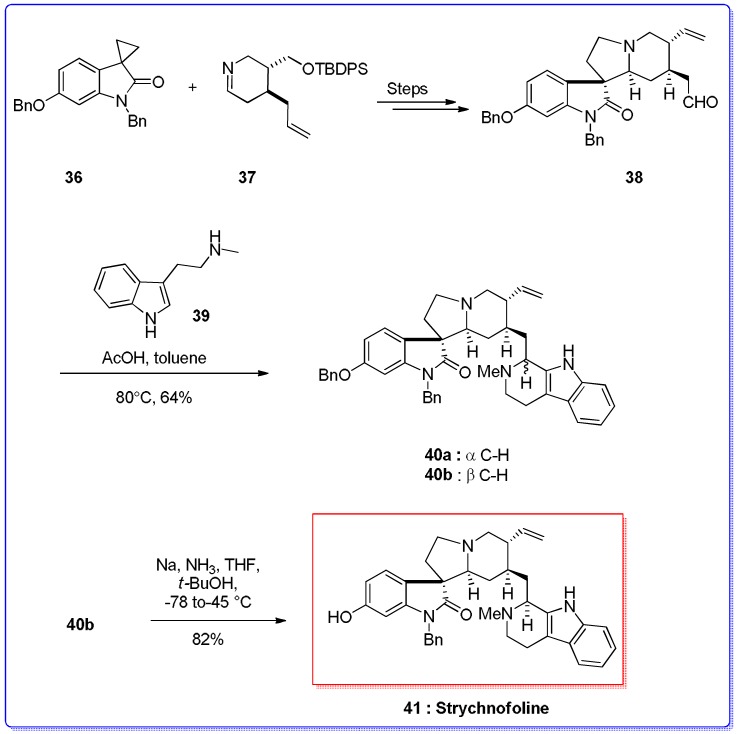
Total synthesis of strychnofoline **41**.

In this route, for the synthesis of (±)-strychnofoline, the reaction of **36** and imine **37** occurred and after several steps afforded aldehyde **38**. With this pathway, PSR [117] between aldehyde **38** and *N*-methyl-tryptamine **39** using acetic acid in toluene at 80 °C gave a diastereomeric mixture of products **40a** and corresponding **40b** nonselectively (1.5:1) in a combined yield of 64% [118,119]. Remarkably, deprotection of **40b** gave (±)-strychnofoline **41** in 82% yield (Scheme 8) [116].

Eudistomins, extracted by Rinehart and co-workers in 1984 [120,121,122] from a *Caribbean tunicate* (*Eudistoma oliVaceum*), are members of the tetrahydro-β-carboline group of marine alkaloids. Among eudistomins, eudistomin C, E, K, L, and F, containing the hither to unknown oxathiazepine ring, demonstrated highly significant antiviral properties against both RNA and DNA viruses as well as antimicrobial and antitumor properties [122,123].

Fukuyama and co-workers in 2005 demonstrated the stereo controlled total synthesis of (−)-eudistomin C **46** which relied on the development of the Brønsted acid-mediated diastereoselective PSR and the unprecedented production of an unusual oxathiazepine ring. The 18-step reaction with an overall yield of 7.7% permitted the gram-scale formation of eudistomin C to be performed as well as diverse derivatives.

**Scheme 9:**
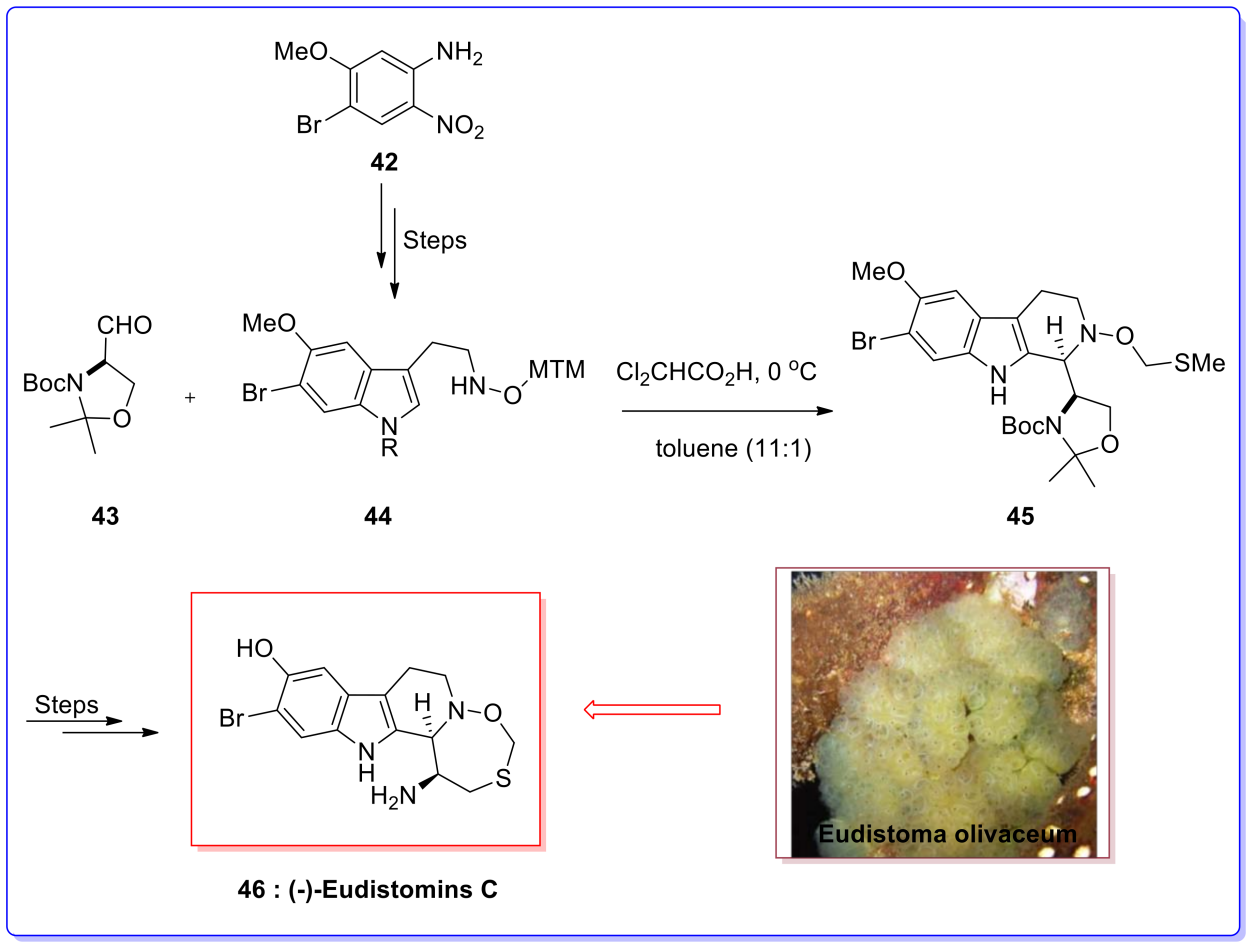
Total synthesis of eudistomins C **46.**

In this method, for the synthesis of (−)-eudistomin C **46**, at first, the synthesis of the indole part [124] initiated from nitroaniline **42**, was easily synthesized from *m*-anisidine in four stags [124]. The diastereoselective production of tetrahydro-β-carboline could be achieved by the PSR [125] of the tryptamine derivative and Garner aldehyde **43** [126,127]. The researchers focused their attention on the critical PSR of Garner aldehyde **43**. Meanwhile, a first effort using a model substrate of **44** lacking the bromo and methoxy substituents afforded the unexpected diastereomer as the major product (3:1) under conventional reaction conditions (trifluoro acetic acid in dichloromethane, −78 °C), They examined a wide range of acid catalysts and solvents. Astonishingly, they realized by using a catalytic quantity of dichloro-acetic acid or chloro-acetic acid in toluene the reaction proceeded smoothly at 0 °C to give the corresponding diastereomer **45** with excellent selectivity (11:1). After several steps, compound **45** was converted into the natural product (−)-eudistomin C **46** (Scheme 9) [128].

Mitragynine **52** was extracted from *Mitragyne speciosa Korth* [129,130] and used as a substitute for opioids of painin in Thailand. In 1965, the X-ray crystal structure of the hydroiodide salt of mitragynine was obtained for its certain structural elucidation [131]. However, mitragynine was the main alkaloid from the extract of *Mitragyne speciosa*, a more careful examination demonstrated that a more significant alkaloid, was existent in the mature leaves of *M. speciosa* (Thailand). In addition, this hydroxyl derivative could be provided from the oxidation reaction of mitragynine with iodobenzene di-acetate [132]. Fascinatingly, the methoxyl functional substituent was known, being required for its analgesic property [133].

The alkaloid 9-methoxy-geissoschizol [132] was extracted from the bark of *Strychnos guianensis* [134], that is known in the basin of the upper and middle Orinoco rivers and throughout the Amazon basin. The crude extracts from the root and stem bark demonstrated a muscle relaxant property [135]. The related 9-methoxy-*N*_b_-methylgeissoschizol **54** that is a quaternary indole alkaloid was recognized later [136].

An asymmetric strategy for the formation of 4-methoxy tryptophan was accomplished through a regiospecific Larock hetero annulation and used for the initial total synthesis of 9-methoxy geissoschizol **53**, 9-methoxy-*N*_b_-methylgeissoschizol **54**, and the total synthesis of mitragynine **52**, starting from 4-methoxy-d-tryptophan **47**. The enantioselective PSR and Ni(COD)_2_-catalyzed cyclization reaction acted as main stages to setup the stereochemistry at C(3) and C(15) in these indole alkaloids.

**Scheme 10:**
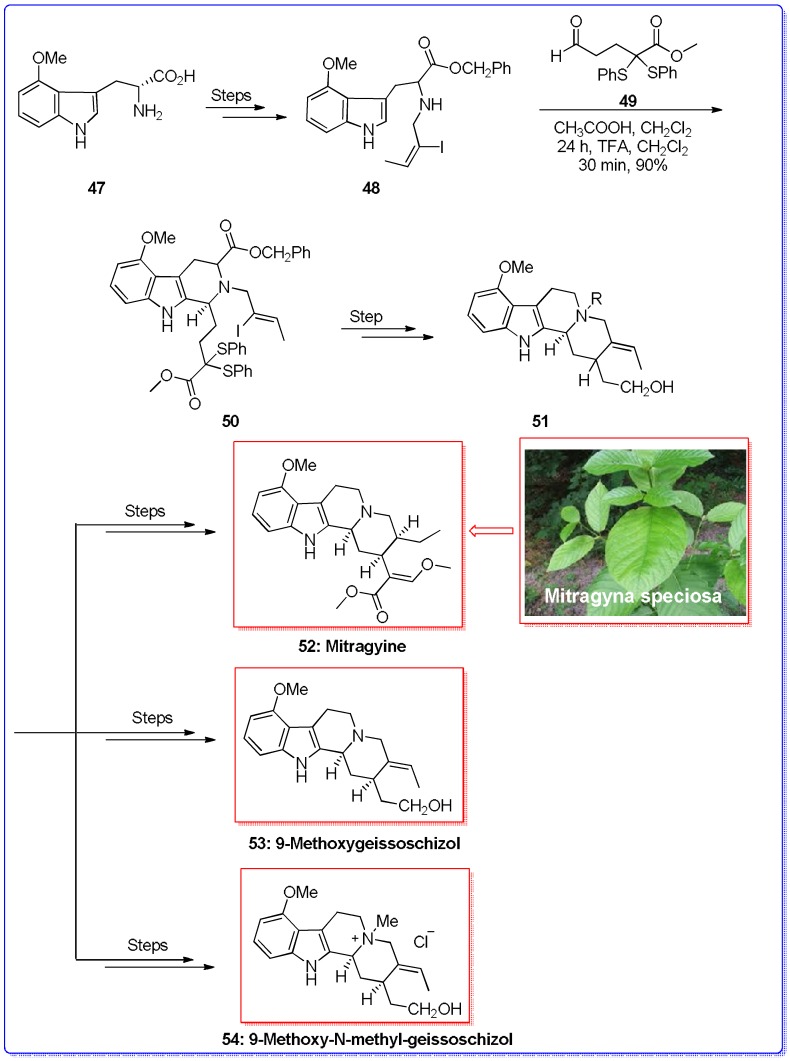
Total synthesis of mitragyine **52**, 9-methoxygeissoschizol **53,** and 9-Methoxy-*N*_b_-methylgeissoschizol **54**.

Total synthesis of natural products **52**, **53,** and **54** were initiated from 4-methoxytryptophan which after several steps afforded the secondary amine **48**. The corresponding stereocenter at C-3 was accomplished through the enantioselective PSR [4] of the secondary amine **48** and the aldehyde [137] **49** to supply the tetrahydro-β-carboline **50**. Next, this diester **50** was transformed into the corresponding α,β-unsaturated ester **51** in 64% overall yield through a number of normal conversions comprising elimination of one equiv of thiophenol from tetrahydro-β-carboline **50**, followed by an oxidation reaction with *meta*-chloroperoxy benzoic acid and a sulfoxide removal sequence [137,138]. Next, after several steps, 9-methoxygeissoschizol **53** was synthesized in 90% yield. In addition, 9-methoxy-*N*_b_-methylgeissoschizol **54** was formed through the *N*_b_-methylation of **53** with methyl iodide followed by exchange of the iodide to the chloride by silver chloride. The ^13^C-NMR data of synthetic (+)-**54** was in agreement with those determined for the naturally occurring compound [134,136]. Besides this, through another approach from α,β-unsaturated ester **51**, after several steps, mitragynine **52** was synthesized (Scheme 10) [139].

Yohimbine **59**, a significant member of the monoterpenoid indole alkaloids, belongs to a large group of naturally occurring compounds which shows synthetically challenging structures showing different biological properties [140,141]. The total synthesis of (+)-yohimbine was accomplished in 11 steps and 14% overall yield in 2008 by Jacobsen and co-workers [142]. The absolute configuration was developed by an extremely asymmetric thiourea-mediated acyl-PSR, and the remaining four stereocenters were set concurrently in a substrate-controlled intramolecular Diels-Alder reaction.

**Scheme 11:**
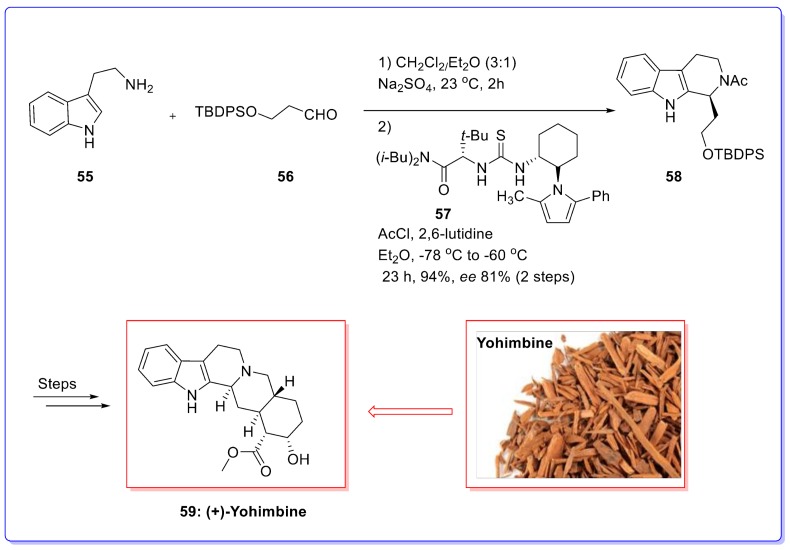
Total synthesis of yohimbine **59.**

In this pathway, total synthesis of (+)-yohimbine was begun with the formation of *N*-acetyltetrahydro-β-carboline **58** through the acyl-PSR [143]. Condensation of tryptamine **55** with aldehyde **56** [144], and the resultant imine with acetylchloride and 2,6-lutidine using thiourea catalyst **57** (10 mol%) gave **58** in 81% yield and 94% *ee* on a gram scale. After several steps, (+)-yohimbine **59** was provided in 14% overall yield (Scheme 11) [142].

The deplancheine-typetetracyclic indole alkaloid arboricine **65**, extracted from the leaves of *Kopsiaarborea* by Kam and co-workers in 2009, exhibited an adequate ability to reverse multi-drug resistance in vincristine-resistant KB(VJ300) cells [145].

Hiemstra and co-workers in 2009 reported, a significant six-step synthesis initiated from tryptamine affording the tetracyclic-carboline arboricine in 33% overall yield through an asymmetric organocatalytic PSR and intramolecular palladium(0)-mediated vinyliodide-enolate coupling as the main stages. Another three-step pathway initiated from tryptamine afforded arboricine in an overall yield of 35% but lower *ee*.

The synthesis initiated with the tryptamine [146], generated in one-step through alkylation reaction of tryptamine and *Z*-2-iodo-2-butene-1-olmesylate in 84% yield (not shown in the Scheme) [146] PS condensation reaction of **60** with aldehyde **61a** catalyzed by (*R*)-3,3′-triphenylsilyl-binolphosphoricacid **63a** ((*R*)-binol-PA, 5 mol%) afforded β-carboline (S)-**62a** together with aminal **64** in 55% yield and a 75/25 ratio, respectively, and an unacceptable 38% *ee* for both (*S*)-**62a** and **64**, as identified by chiral HPLC. Happily, masking of ketone **61a** as the dioxolane **61b** not only evaded aminal construction therefore solely affording (*S*)-**62b** in 81% yield but also increased the *ee* to 78%. It is significant that installing the acetal masking substituent, that is quite remote from the iminium intermediate, increases both the enantioselectivity and the rate of the PSR using lower catalyst loadings down to 1%. The mildness of the method is underscored by the fact that the dioxolane-protected ketone stayed unaffected. Clearly, (*R*)-**62b** has been produced initiated from (*S*)-binol-PA. Remarkably, the best *ee* was obtained using the sterically somewhat more demanding catalyst (*R*)-H_8_-binol-PA **63b**, which afforded (S)-**62b** in 86% yield and 89% *ee*. Scaling up the reaction to 5 mmol only required 1 mol% of catalyst **63a** giving (*S*)-**62b** in an extracted yield of 92% and 78% *ee*.

To circumvent the probable racemization through acid-mediated scission of the bond between the enantioselective carbon atom and *N*_b_ [147] during the hydrolysis of the acetal scaffold, a Boc-masking substituent was achieved on the indole. Reaction of **62b** with Boc_2_O and DMAP followed using diluted hydrochloric acid in acetone afforded the corresponding ketone which in two steps gave (−)-arboricine **65** in 81%. Synthetic arboricine was enantio pure by HPLC (method A).

**Scheme 12:**
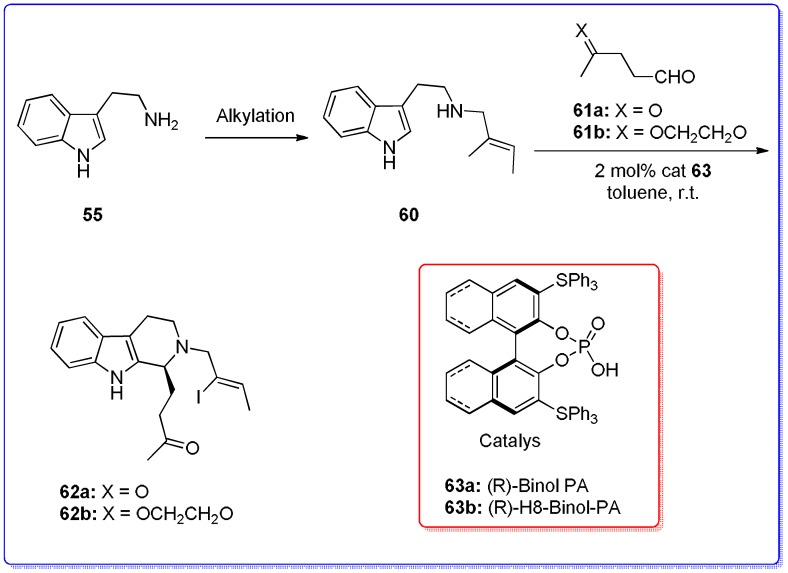
PSR in the presence of **63** as a catalyst.

**Scheme 13:**
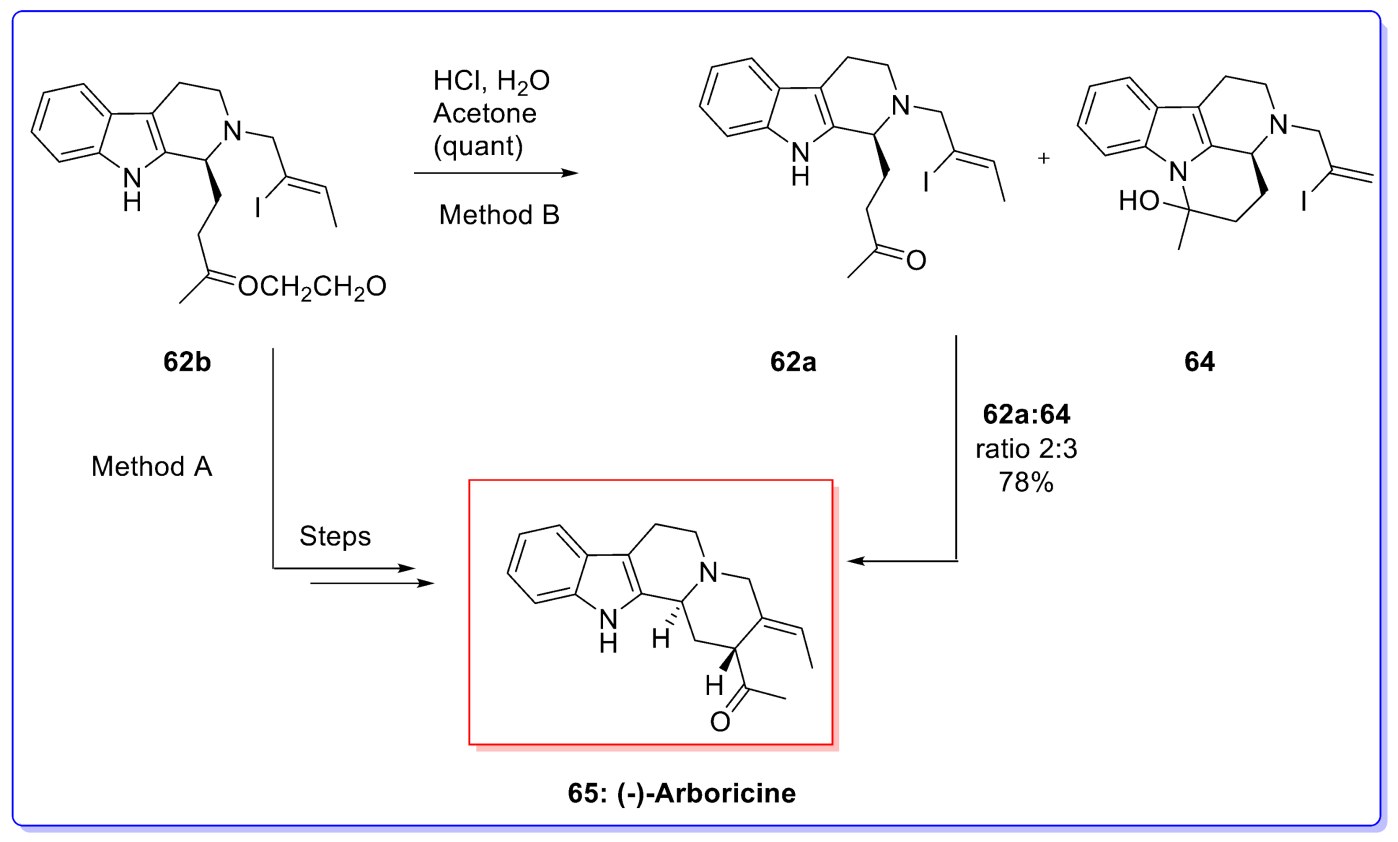
Total synthesis of tetracyclicindole alkaloid (−)-arboricine **65.**

In another pathway, dissolving **62b** in dilute hydrochloric acid in acetone afforded quantitatively a mixture of **62a** together with aminal **64** in a 2/3 ratio, respectively. Gratifyingly, based on the slightly basic conditions of the final palladium(0)-mediated cyclization, an equilibrium between **62a** and **64** existed permitted a significant and diastereoselective transformation to arboricine **65** in a yield of 78%, although *ee* dropped from 86% to 65% (method B) (Scheme 12 and Scheme 13) [148].

Henrycinols A and B, two indole alkaloids, extracted from *Melodinus henryi* CRAIB of *Apocynaceae* genus by Zhang and co-workers [149]. These alkaloids belong to the class of 1,2,3,4-tetrahydro-β-carbolines, a structural scaffold, that is, abundant in a range of indole alkaloids [150]. Structurally henrycinols A and B differ from simple 1,2,3,4-tetrahydro-β-carbolines with the presence of two hydroxyl substituent on the D ring of the alkaloid. The total synthesis of indole alkaloids henrycinol A and B were performed initiating from l-tryptophan methyl ester. The main step is a stereochemically flexible PSR provided by the presence or absence of an *N*-allyl substituent in the tryptophan precursor. The natural products henrycinol A and B were obtained in satisfactory overall yield in eight and nine steps, respectively. Therefore, the synthetic sequence starting with the PSR of the desired aldehyde [151,152] obtained from (−)-2,3-*O*-isopropylidene-d-threitol **66** with l-tryptophan methylester **67** gave a separable mixture of 1,3-*cis*tetrahydro-β-carboline **69α** as the main product in 50% yield and 1,3-*trans* tetrahydro-β-carboline **69β** in 18% yield [149].

Since the naturally occurring compound included the *trans* tetrahydro-β-carboline isomer, it was posed with the challenge of procuring the needed 1,3-*trans*-1,2,3,4-tetrahydro-β-carboline in excellent yield in the PSR. Pioneering work by Cook’s group [153,154,155] established the transformation of 1,3-*cis* to 1,3-*trans* products in the PSR of tryptophan methylester with benzaldehyde. They detected that the reaction of *N*-functionalized tryptophan esters rendered the 1,3-*trans*-1,2,3,4-tetrahydro-β-carbolines using non-acidic conditions, whereas simple tryptophan esters without substitution gave the 1,3-*cis* carbolines under acidic conditions. Considering these results, it was reasoned that the PSR of *N*-allyl-l-tryptophan methylester **68** with the corresponding aldehyde should provide the 1,3-*trans*-1,2,3,4-tetrahydro-β-carboline **70b**. Actually, this was known to be the case, and the reaction between *N*-allyl-l-tryptophan methylester **68** and the corresponding aldehyde obtained from (−)-2,3-*O*-isopropylidene-d-threitol **66**), gave the 1,3-*trans*-1,2,3,4-tetrahydro-β-carboline **70β** as the main product in 79% yield. After several steps, the 1,3-*trans*-1,2,3,4-tetrahydro-β-carboline **70β** gave henrycinol A **71** in 78% yield.

**Scheme 14:**
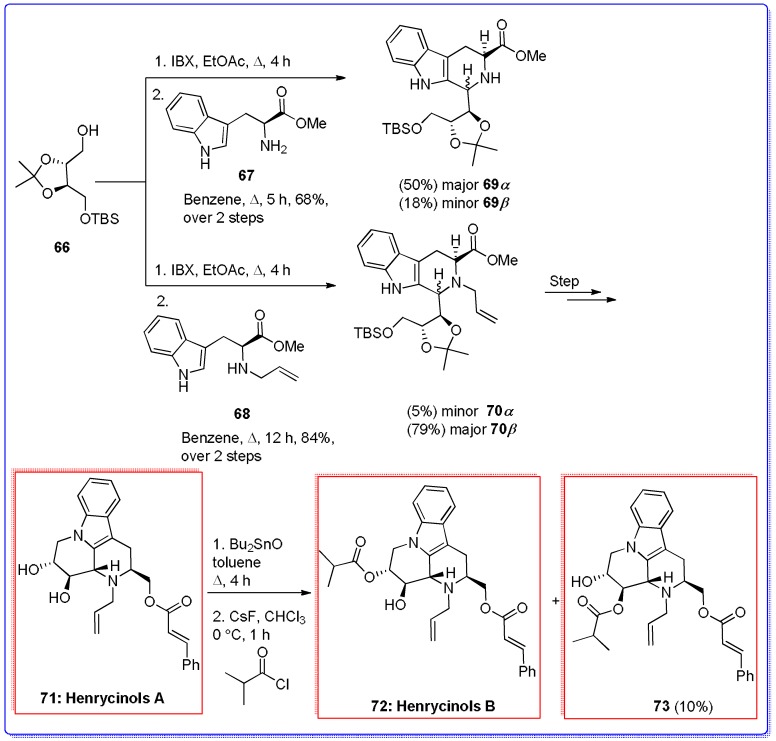
Total synthesis of natural products henrycinols A **71** and B **72**.

The stereochemistry of the freshly provided stereogenic center in the PSR and the structure of the natural product was definitely confirmed by X-ray crystal structure analysis of henrycinol A **71**. Furthermore, reaction between henrycinol A **71** and isobutyrylchloride using Bu_2_SnO and CsF [156,157] gave henrycinol B **72** in 24% yield, the regioisomer **73** in 10% yield and the recovered initiating compound henrycinol A **71** in 47% yield. Significantly, stereoselective first total synthesis of the indole alkaloids henrycinol A **71** and B **72** were performed from *N*-allyl-l-tryptophan methylester **68** and (−),2,3-*O*-isopropylidene-d-threitolin 34% and 8% overall yields, respectively in eight and nine linear stages. The key conversion was the *trans*-selective construction of 1,3-dialkyl-1,2,3,4-tetrahydro-β-carboline in the Pictet–Spengler cyclisation (Scheme 14) [158].

The remarkable pharmacological activities in cerebral circulation and neuronal homeostasis of eburnamine-vincamine indole-type alkaloids, including vincamine **79a**, eburnamine **79b**, and vinpocetine **79c** [159,160], make them striking products for total synthesis [161]. Until 2014, the most usual method to develop the [ABCD]-ring system of these compounds was to begin from an indole subunit to make the final E-ring [162,163,164]. Significant production of a *cis*-[ABCD]-ring intermediate, the katsubenitrile **78**, is the main stage for synthesis of **79**. The katsubenitrile **78** is a building block in the three usual synthetic pathways, that all apply various methods, for the production of the *cis*-stereocenters of **79**. An efficient synthesis of the katsubenitrile is accomplished through a significant diastereoselective PSR of 3-ethyl-2-hydroxy-1-[2-(1*H*-indol-3-yl)ethyl]-6-oxopiperidine-3-carbonitrile to form the *cis*-[CD] rings in 1-ethyl-4-oxo-1,2,3,4,6,7,12,12β- octahydroindolo[2,3-α]quinolizine-1-carbonitrile as the main stages.

**Scheme 15:**
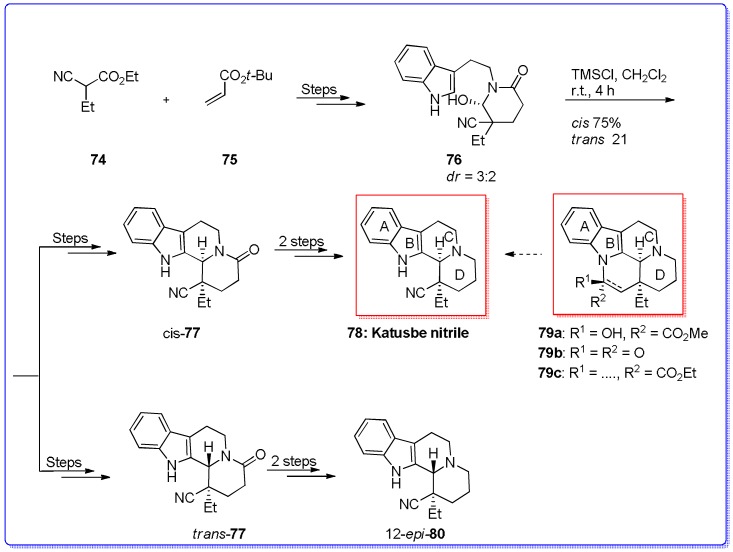
Total synthesis of katusbenitrile **78** using PSR as key step.

In this approach, total synthesis of katsubenitrile **78** was initiated from the readily available ethyl-2-cyanobutanoate [165] and *tert*-butylacrylate which after several steps gave the *N*,*O*-hemiacetal **76** as an inseparable 3:2 mixture of epimers. Next, the PSR of **76** was explored to construct the tetracyclic compound **77**. Reaction of **76** with trifluoro acetic acid (TFA) in CH_2_Cl_2_ at −55 °C afforded no reaction, while, elevating the reaction temperature to 20 °C afforded the corresponding compound **77** as a 2:1 mixture of epimers. A short investigating action of different acids occurred, and it was recommended that chlorotrimethylsilane was the most effective for control over the diastereoselectivity at ambient temperature, giving **77** in a 96% yield with a diastereomeric ratio of 3.5:1. These epimers of **77** could be easily separated by chromatography. In addition, the extremely crystalline nature of the epimers permitted X-ray crystallographic analysis, thus confirming the stereochemical assignment. Upon two steps, katsubenitrile **78**, a main synthetic intermediate for eburnamine-vincamine alkaloids, was provided in satisfactory yields (2:68%, 12-*epi*-2:65%) (Scheme 15) [166].

**Scheme 16:**
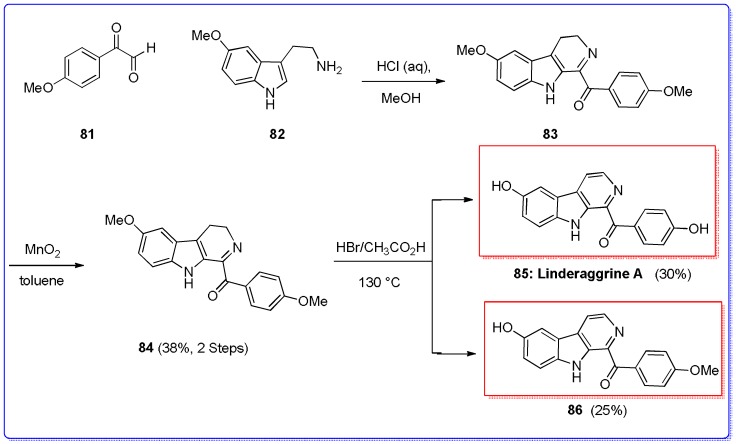
Synthesis of linderaggrine A **85** and mono-demethylation product **86**.

**Scheme 17:**
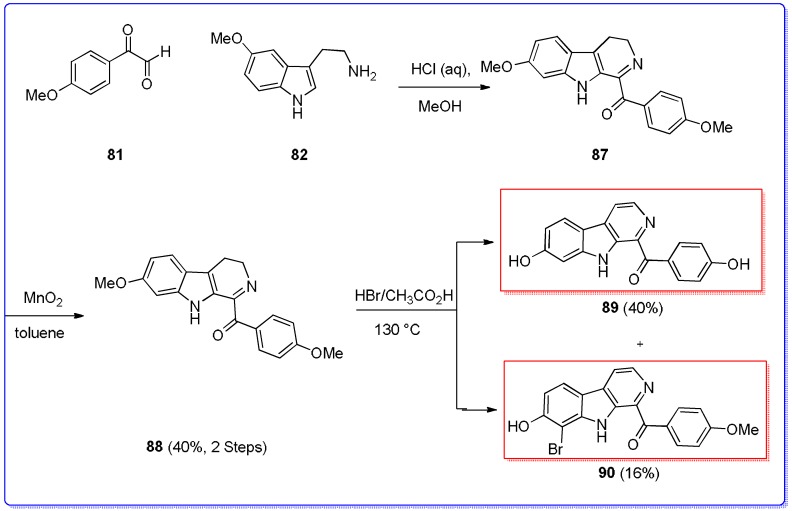
Synthesis of product **89**, the isomer of linderaggrine A, and 8-brominated product **90.**

Lindera aggregate is extensively dispersed in China and widely employed in traditional Chinese medicine for the treatment of a number of physiological symptoms [167]. Pharmacological investigating actions on *L. aggregata* (*Lauraceae*) demonstrated diverse important bioactivities, comprising super oxide anion radicals avenging, and protection against post-ischemic myocardial dysfunction, as well as slowing down the progression of diabetic nephropathy in db/db mice [168,169,170]. Linderaggrine A **85** is a β-carboline alkaloid, from the roots of *L. aggregata*. β-Carboline alkaloids are a predominant class of biologically active naturally occurring compounds having an extensive range of pharmacological and structural diversity [171,172,173]. The successful construction of linderaggrine A **85** and **89** gave unambiguous evidence for the determination of the naturally extracted product. Wu and co-workers in 2014 designed synthesis of 1-functionalized β-carbolines, using a single-step PSR [174]. The synthetic approaches employed to provide linderaggrine A **85** and its isomer by single-step PSR of 5-methoxytryptamine or 6-methoxy tryptamine with *p*-methoxy phenyl glyoxal were demonstrated. The precursors 5-methoxy tryptamine or 6-methoxy tryptamine and *p*-methoxy phenyl glyoxal were reacted by PSR to result in a mixture of dihydro-β-carbolines **83** or **87** and β-carbolines **84** or **88**. To increase the yield of this conversion, MnO assisted dehydrogenation provided the aromatic β-carbolines **84** or **88** in satisfactory yields (38% and 40%, respectively). The deprotection of the methyl substituent in both phenyl rings was examined by either HCl or AlCl_3_; though, only one of the methyl substituents could be disintegrated. Thus, the hydrobromic acid–acetic acid pair was applied to deprotect the two methyl substituents in one step with satisfactory yields (30% for **85** and 40% for **89**, respectively). But, there is a slight difference between the synthetic products of **85** and **89**. In the deprotection reaction of **88** to **89**, 8-brominated product **90** was afforded because of the bromine provided from the decomposition of HBr. In contrast, demethylation of β-carboline **84** merely afforded the mono-demethylation product **85** and the desired di-demethylation product **86**. The bio-activity consequences demonstrated the application of the roots of *L. aggregata* as herbal medicines in the reaction of inflammatory diseases, and linderaggrine A **85** may be potential in examining novel anti-inflammatory lead drugs (Scheme 16 and Scheme 17) [175].

**Scheme 18:**
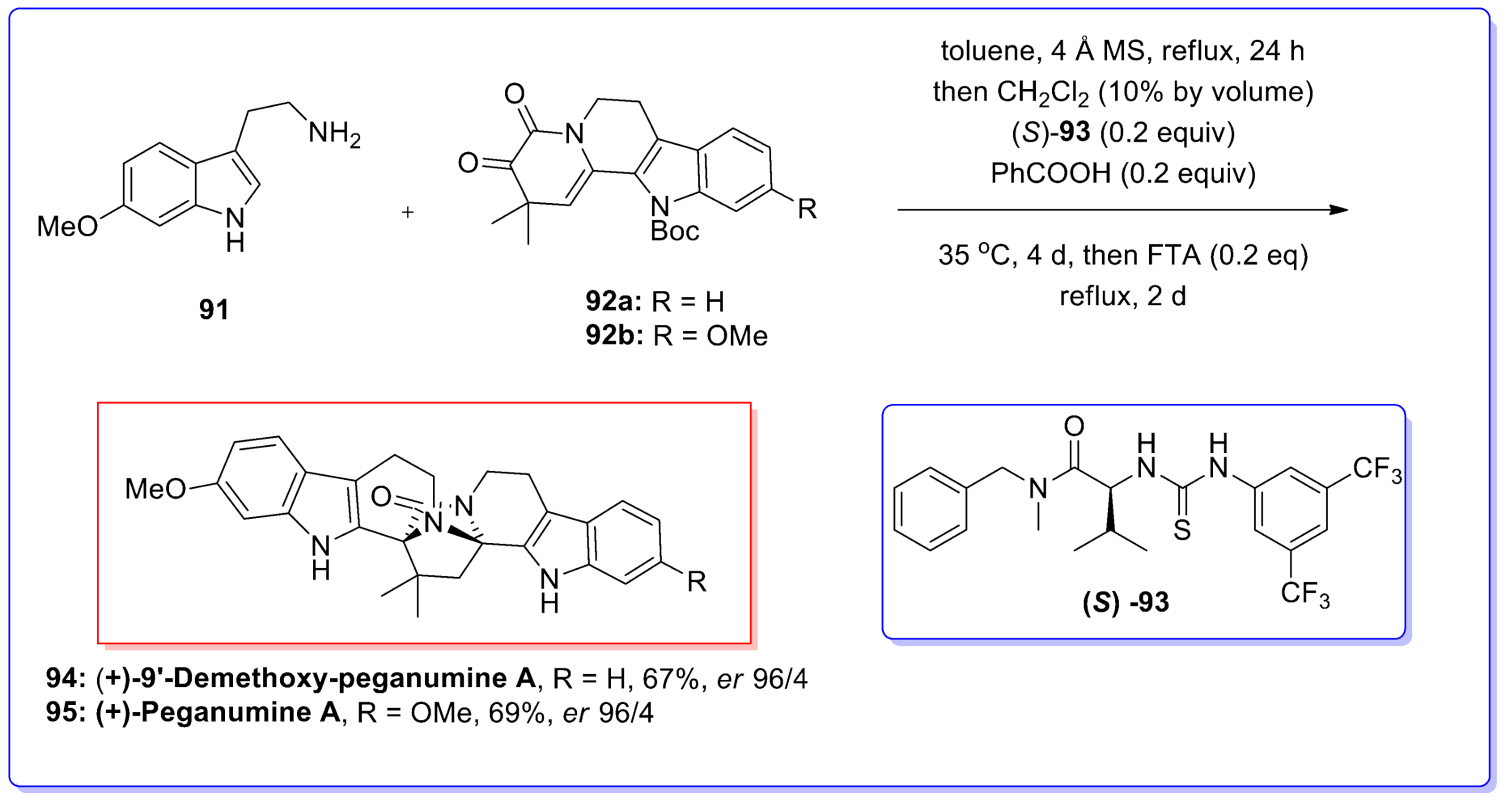
Thiourea-catalyzed enantioselective synthesis of (+)–peganumine A **95** by PSR as the key step.

Peganumine A **94**, a dimerictetrahydro-β-carboline alkaloid, was extracted by Li, Hua and co-workers in 2014 from the seeds of *Peganum harmala* L [176]. Its octacyclic structure having a distinctive 3,9-diazatetracyclo-[6.5.2.00] [176,177,178,179] pentadec-2-one moiety has been unprecedented. It demonstrated importantly the toxic property against MCF-7, PC-3, Hep G2 cells and selective influence on HL-60 cells. A gram-scale asymmetric total synthesis of (+)-peganumine A was achieved in seven steps from market purchasable 6-methoxytryptamine. Key stages comprised a Liebes kind-Srogl cross coupling; a one-pot production of the tetracyclic scaffold from an ω-isocyano-γ-oxo-aldehyde through a sequence of an unprecedented carbon–carbon bond providing lactamization and trans annular condensation reaction; as well as a one-pot organo-catalytic method merging two a chiral building blocks into an octacyclic structure through a sequence of asymmetric PSR and by a trans annular cyclization reaction. This last reaction generated two spiro cycle derivatives and α-2,7-diazabicyclo[2.2.1]heptan-3-one part along with excellent control of both the absolute and relative stereochemistry of the two freshly generated quaternary stereocenters. Generally, (+)-peganumine A **95** was formed in seven steps with 33% overall yield (*er* 96/4) from the market purchasable 6-methoxytryptamine; the application of this synthetic method being accepted. In the following, the conditions to achieve a catalytic enantioselective synthesis of (+)-peganumine A were examined. The reaction of **91** and **92a** using chiral phosphoric acid (TRIP) indeed gave 9′-demethoxy-peganumine A **94**, although with poor yield (7%) and *ee* (*er* 64.5/35.5) [180]. Employing Jacobsen’s chiral thio urea catalyst (S)-**93** was known to be more satisfactory [181]. Then, trifluoro acetic acid was added and the reaction mixture was refluxed for an additional two days to give (+)-9′-demethoxy-peganumine A **94** in 67% yield with *er* of 96/4. Applying PhCOOH as co-catalyst was of greatest significance for the enantioselectivity of the reaction since employing acetic acid in place of PhCOOH under otherwise similar conditions afforded compound **94** (75% yield) with significantly decreased *ee* (*er* 72/28). Condensation reaction of **91** with **92b** gave (+)-peganumine A **95** (69%, *er* 96/4) in which the spectroscopic data were identical in all respects to those reported for the naturally occurring compound. As a result, the natural enantiomer was generated using (*S*)-**93** as catalyst (Scheme 18) [182].

**Scheme 19:**
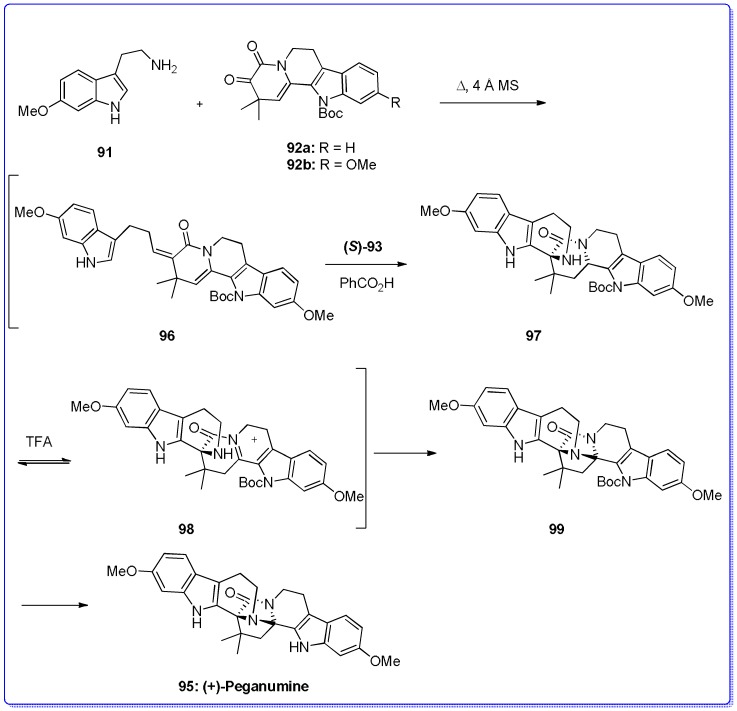
Total synthesis of (+)-peganumine A **95**.

In this route, total synthesis of (+)-peganumine A **95** began with 6-methoxytryptamine. The condensation reaction of amine **91** with α-ketoamide **92b** gave imine **96**, that underwent the asymmetric aza-Friedel-Crafts addition under the effect of the thiourea (*S*)-**93** and PhCOOH to give the enantio enriched **97**. After addition of a catalytic quantity of strong acid (trifluoro acetic acid), enamine-imine tautomerization occurred to form **98**, that, after stereo specific *trans* annular addition of the secondary amine to iminium, provided octacycle **99**. Elimination of *N*-Boc provided the natural product **95**. Two quaternary stereocenters were made from two a chiral building blocks with excellent control of both *de* and *ee*s (Scheme 19) [182].

The genus Kopsia, that belongs to the family *Apocynaceae*, is a rich source of mono terpenoid indole alkaloids containing extensive series of biological properties and structural diversity [183]. Takayama completed the structure clarification of different unique monoterpenoid indole alkaloids from *Kopsia arborea*, native to the Yunnan Province in China, [184,185] including an intriguing significant alkaloid named kopsiyunnanine K **104**, that has an unprecedented azepine-fused tetrahydro-β-carboline ring moiety. A monoterpenoid indole alkaloid, kopsiyunnanine K **104**, was extracted from *Kopsia arborea*. Its fascinating rearranged structure and absolute configuration, inferred from spectral data, and the probable biosynthetic route were identified on the basis of a 13-step enantioselective total synthesis.

**Scheme 20:**
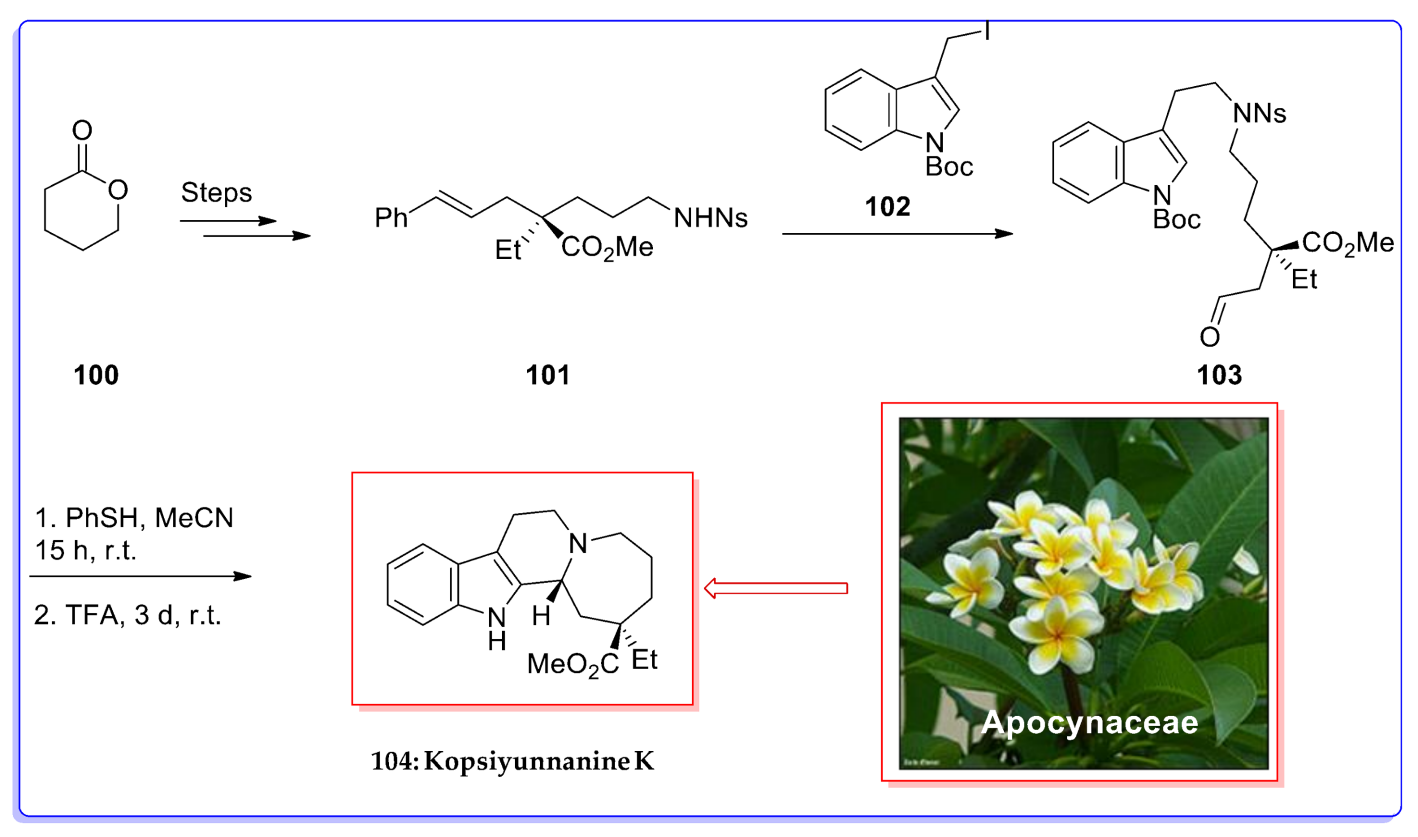
Total synthesis of kopsiyunnanine K **104**.

Takayama and co-workers in 2016 reported the enantioselective total synthesis of kopsiyunnanine K **104** through an enantioselective Ireland–Claisen rearrangement and an intramolecular diastereoselective PSR, and exhibited its significant rearranged framework and absolute configuration. Total synthesis of kopsiyunnanine K **104**, was initiated from market purchasable valerolactone **100**, and after several steps comprising oxidation, Mitsonubu reaction, ozonolysis, and alkylation afforded aldehyde **103**. Next, deprotection of the N_s_ group on the N_b_ position in **103** followed through intramolecular diastereoselective PSR of the obtained amine using trifluoroacetic acid (TFA) afforded kopsiyunnanine K **104** as a single diastereomer in a measurable yield. Recrystallization of the product gave optically pure **104**. Noticeably, the structure and the 16*R*, 20*R* configuration of synthetic **104** were confirmed by X-ray crystallographic analysis (Scheme 20) [186].

Spirooxindole alkaloids are interesting and challenging synthetic products, that have extremely complicated building blocks combined with favorable properties in numerous therapeutic areas [187,188]. Illustrative spirooxindole alkaloids contain trychnofoline **109**, spirotry prostatins [115], palmirine [189], citrinadins [190], gelsemine [191], and cyclopiamines [192]. Amongst these attractive molecules, **109** seems to be a significant target for chemical synthesis and biological examination. It was extracted from the leaves of *Strychnos usambarensis* by Angenot and co-workers in 1978, and exhibited extremely promising antimitotic property against cultures of Ehrlich tumor cells and mouse melanoma. A striking synthesis of (±)-**109** was shown by the Carreira group, in 2002, utilizing an elegant, extremely diastereoselective cyclopropane ring expansion method [116]. The five stereocentres and the unique spiro[pyrrolidin-3,3′-oxindole]scaffold show a substantial challenge for its synthesis.

Strychnofoline is a Strychnos alkaloid that has a significant spirooxindole framework and has a significant anticancer property. Xu and co-workers in 2018, for the first time, demonstrated the asymmetric synthesis of strychnofoline proceeding in only nine steps from market purchasable 6-methoxytryptamine.This method is highlighted by a one-pot, catalytic enantioselective production of the quinolizidine intermediate **108**. The efficacy of the synthesis derives from the use of two sequential conversion stages in the catalytic enantioselective production of the spiro[pyrrolidine-3,3′-oxindole] scaffold in a simple method. Remarkably, the β-carboline framework could be generated through a late stage PSR. This pathway was performed through sequential acylation/enantioselective Michael addition/PSR/oxidative rearrangement.

**Scheme 21:**
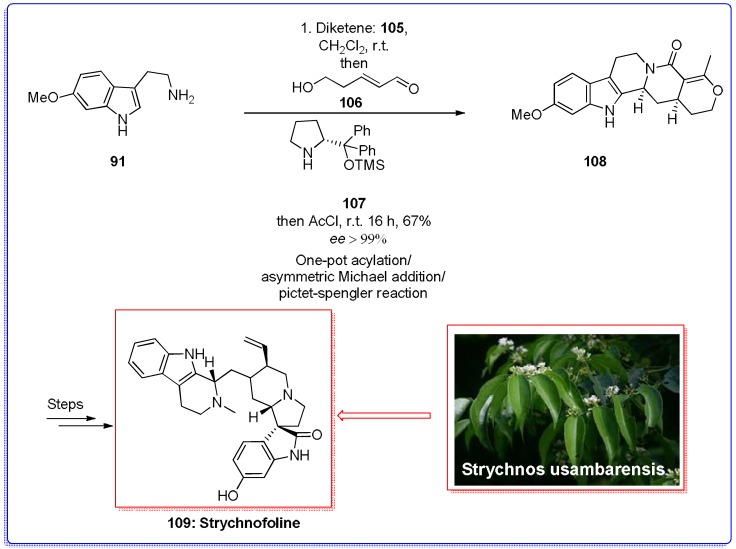
Total synthesis of strychnofoline **109.**

Total synthesis of strychnofoline was initiated from market purchasable 6-methoxytryptamine **91**. By sequentially adding **91**; diketene **105**, acrolein derivative **106**; organocatalyst **107** (Hayashi-Jorgensen catalyst); and acyl chloride to the reaction mixture, they were able to obtain the quinolizidine derivative **108** in satisfactory yield with high *ee* (67% yield, *ee* >99%). After several steps, total synthesis of the anti-tumor alkaloids trychnofoline **109** was completed and the synthesized material, showed equal spectroscopic and analytical properties to that demonstrated for the naturally occurring compound [113,116]. A useful construction of **109** will be of great assistance in addressing its therapeutic promise (Scheme 21) [193].

The C-19 methyl functionalized macroline/sarpagine and ajmaline alkaloids are an emerging group of biosynthetically related indole alkaloids, some of which have historical importance [194], and were principally extracted from different medicinal plants of the *Apocynaceae* group. Most of these alkaloids were not examined for their biological property, probably, because of the paucity of extracted material. Macrocarpines B were extracted from the stem bark of *Alstonia macrophylla* by Kam [195]. Talcarpine **117**, extracted from *Alstonia macrophylla* and *Pleiocarpa* talbotii, showed antimalarial properties [71]. *N*(4)-Methyl-*N*(4), 21-secotalpi-nine **118**, extracted from *Pleiocarpa talbotii*, and *Alstonia angustifolia*, exhibited remarkable anti-leishmanial properties [195,196,197].

The majority of these alkaloids have the β-methyl configuration at C-19, a few contain the α C-19 methyl function (for example dihydroperaksine **119**, also found as dihydrovomifoline and deoxyperaksine) [198]. All of these alkaloids contain either a *N*_a_-methyl or *N*_a_-hydrogen functionalized indole nitrogen atom. Also, the *N*_b_-nitrogen atom differs in the pattern of substitution. Furthermore, all of these alkaloids include a 6 or 7 quaternary center showing different substitution patterns and configurations that render the synthesis of these alkaloids of interest.

**Scheme 22:**
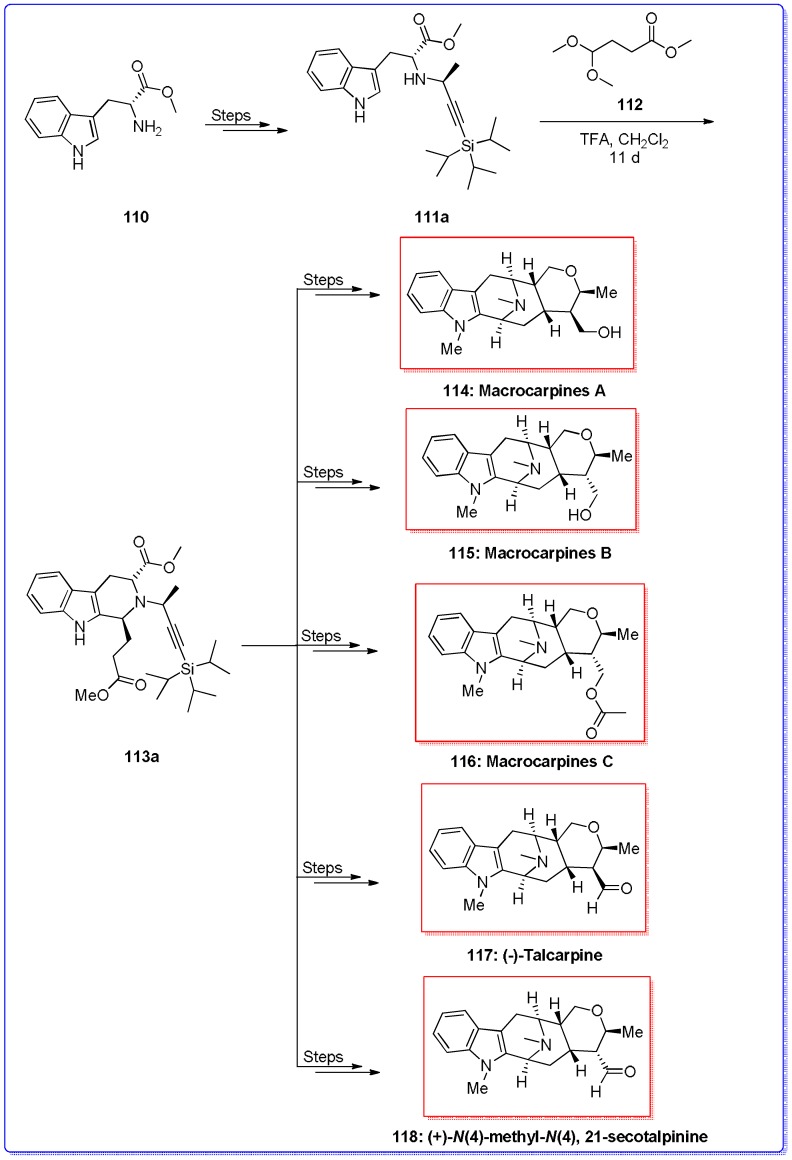
Total synthesis of (−)-macrocarpine A **114**, (−)-macrocarpine B **115**, (−)-macrocarpine C **116**, (−)-talcarpine **117,** and (+)-*N*(4)-methyl-*N*(4),21-secotalpinine **118.**

Extension of the enantioselective PSR to bulkier *N*_b_-alkylated tryptophan led to an increased stereospecific admittance to the key bi-cycle [3.3.1] nonane unit of bioactive C-19 methyl functionalized sarpagine/macroline/ajmaline indole alkaloids having high diastereoselectivity through internal enantioselective induction. Full stereo control of the C-19 methyl function in either the α-or β-configuration has been accomplished that allows the total synthesis of any member from this class of thirty alkaloids. In 2017, the total synthesis of macrocarpines (A-C) **114**, **115**, **116**, talcarpine **117**, *N*(4)-methyl-*N*(4), 21-secotalpinine **118**, dihydro-peraksine **119**, and deoxyperaksine **120** was reported. In this route, the total synthesis was initiated from market accessible d-(+)-tryptophan **110** and the optically pure ethinyl tosylates which was transformed to compound **111**. After several steps, the *N*_b_-alkylated intermediate **111** reacted with the actetal **112** based on the thermodynamically controlled conditions of the enantioselective Pictet–Spengler condensation to supply the corresponding *trans* diester **113a** in high yield and >95:5 *de*. After several steps and by different routes natural products (−)-macrocarpine A **114**, (−)-macrocarpine B **115**, (−)-macrocarpine C **116**, (−)-talcarpine **117** and (+)-*N*(4)-methyl-*N*(4), 21-secotalpinine **118** were synthesized (Scheme 22).

Upon completion of the total synthesis of the C-19 β-methyl functionalized macroline related alkaloids; **114**–**118**, the focus changed to the synthesis of C-19 α-methyl functionalized sarpagine alkaloids (+)-dihydroperaksine **119**, and (−)-deoxyperaksine **120**. The *trans*-diester **113b** was accessed through the approach demonstrated. After several steps, (+)-dihydroperaksine **119** was obtained. The optical rotation and spectral data for this synthetic (+)-dihydroperaksine **119** were in full agreement with the values in the literature [198]. Also, on the other hand, after several steps, (−)-deoxyperaksine **120** was formed.

**Scheme 23:**
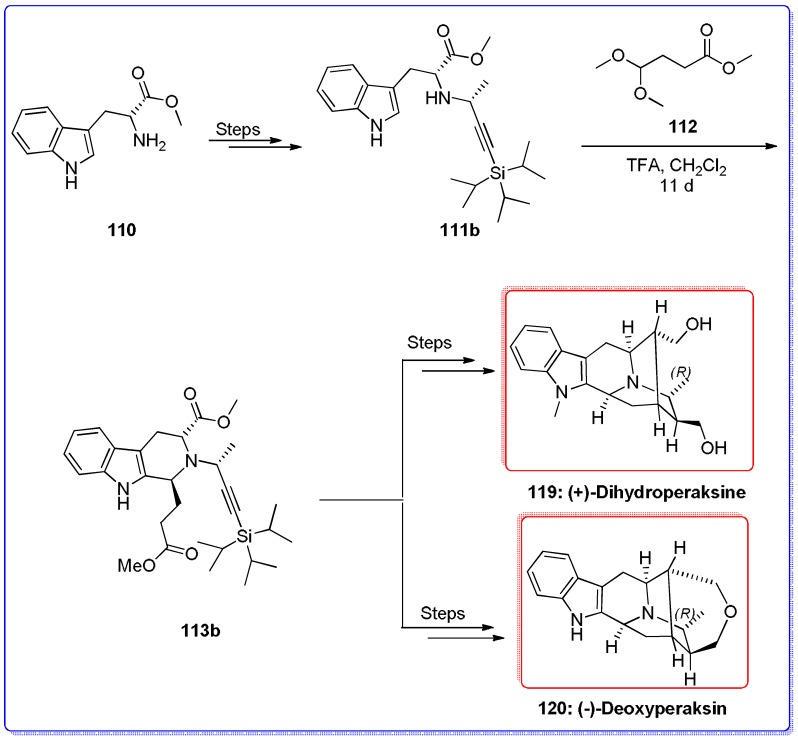
Total synthesis of alkaloids (+)-dihydroperaksine **119** and (−)-deoxyperaksine **120.**

As a result, the initial total synthesis of sarpagine/macroline related alkaloids was accomplished through the expanded and shorter PSR. Furthermore, this route corrects the optical rotation values of (−)-macrocarpine A **114** and (+)-*N*(4)-methyl, *N*(4), 21-secotalpinine **118** demonstrated by others [195]. This route obviously demonstrates that a large group other than the benzyl on the *N*_b_-nitrogen atom of the d-(+)-tryptophan initiating precursor can still give 100% *de* through internal enantioselective induction (Scheme 23) [199].

### 2.2. Phenyl (Tetrahydroisoquinoline) Scaffold

**Scheme 24:**
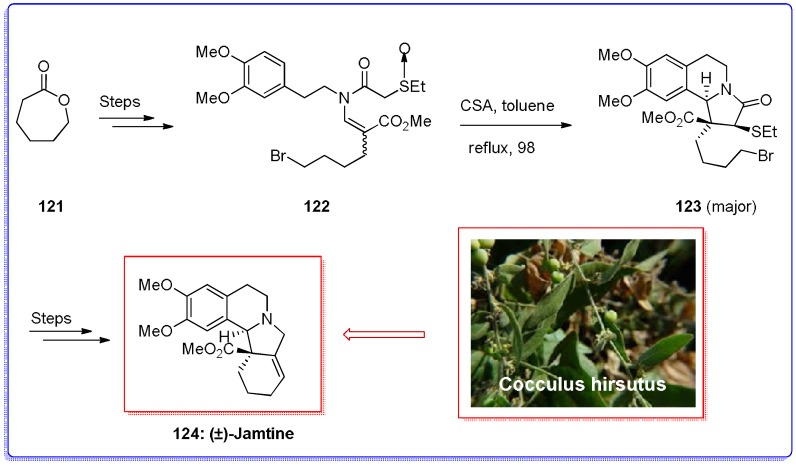
Total synthesis of Jamtine **124**.

Jamtine, one of the significant alkaloids produced by the climbing shrub *Cocculus hirsutus* [200], is known throughout Pakistan and its parts are reputed for their therapeutic properties in folk medicine [201]. Its isolation and structural elucidation, primarily by 2D-NMR spectra, was reported in 1987 [202]. The first total synthesis of (±)-jamtine **124**, a tetrahydroisoquinoline alkaloid reputed for its therapeutic activities, was demonstrated by Padwa and co-workers 2002 [203]. The key stage includes a tandem thionium/*N*-acyliminiumion cyclization using enamidosulfoxide **122**. The cascade method occurs with excellent diastereoselectivity and in high yield. Total synthesis of (±)-jamtine **124** was initiated from commercially available caprolactone **121**, and after several steps gave bromo-enamide **122** as a 4:1(*Z*/*E*) mixture of isomers in excellent yield. Heating the compound **122** with camphor sulfonic acid gave the corresponding tricyclic unit of jamtine in high yield (88%) but as a 5:2:1:1 mixture of diastereomers. The main product obtained corresponded to the corresponding diastereomer **123**. The preferential construction of **123** is consistent with earlier stereo chemical clarifications, [200] demonstrating that a 4π-Nazarov type electro cyclization [204] controls the direction of closure from the α-acylthionium ion intermediate. The sub-sequent PSR includes attack of the proximal aromatic ring from the less hindered side of the iminium ion. After several steps, the first total synthesis of this interesting alkaloid, jamtine **124**, was completed (Scheme 24) [203].

**Scheme 25:**
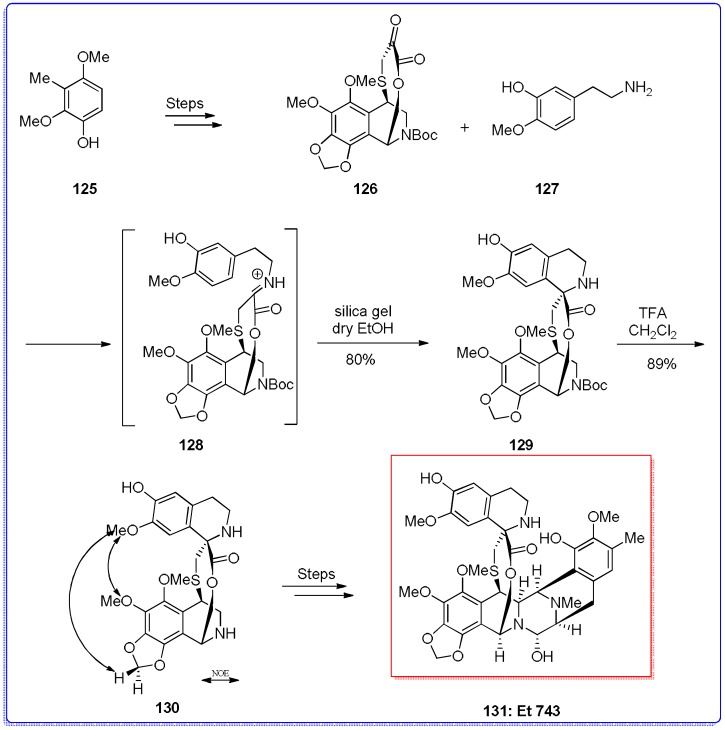
Total synthesis of Et743 **131**.

Ecteinascidin 743 [205,206] (Et743) **131** is one of the significant marine alkaloids, extracted from the *Caribbean tunicate Ecteinascidia turbinata*. However, although extracts from this organism have been investigated since the 1960s, the isolation of pure substances did not occur until 1986 [205,206]. Ecteinascidin 743 [205,206] (Et743) **131**, as a significant antitumor agent is currently undergoing phase II clinical trials and moreover attracting significant attention [207,208,209]. The originality of its architecture, the remarkable biological properties, and its natural scarcity make it attractive for total synthesis [210,211]. In terms of its presentation of the unit pentacyclic A-E ring system, Et743 **131** contains important structural homology to the saframycin group of antibiotics as well as to similar compounds [207,208,209]. The largest variance is that in Et 743, position 4 is a higher oxidation level than in the case of the saframycins. The extra functionality in **131** takes the form of a novel 10-membered ring. This sulfur-comprising macrolactone is itself spiro linked to a tetrahydroisoquinoline. The drug accessibility issue in terms of isolation from natural sources is relatively difficult. Corey and Gin developed first total synthesis of Et 743 [212]. Danishefsky and co-workers in 2002 reported the total synthesis of Et 743 [213]. They reported that Pictet–Spengler cyclization affording spiro product **129** shows excellent stereoselectivity. In this route, the final goal was to attain compounds including **129** and **130** in which the C, D, and E rings of Et 743 were deleted. This group tried to examine the issue of stereoselectivity in the PSR providing **129** (vide infra). After several steps, ketone **126** was formed. Next, the reaction of ketone **126**, accomplished with amine **127** as demonstrated by Corey and Ginina in a more complex setting [212], produced the spiro tetrahydroisoquinoline **129** in an apparently stereo specific method. Because of the rotameric states of **129**, it was difficult to determine the orientation of the spiro attachment. The *N*-Boc linkage was removed using a trifluoro acetic acid reaction, providing the amine **130**. NOE measurements on **130** demonstrated that the orientation at C-1′ corresponded to that required for Et 743. Whether this result is the result of thermodynamic control or reflects some long-range stereochemical preference in mutual presentation of the aromatic sectors of the iminium intermediate (cf. 31) at the kinetic level is not known. In this regard, it is tempting to propose that as the H ring attacks the iminium ion in **128**, the resultant transient electron-deficient cyclohexadienone scaffold is stabilized by stacking to the electron-rich A ring. In this way, the detected sense of face selectivity would be rationalized (Scheme 25) [213].

**Scheme 26:**
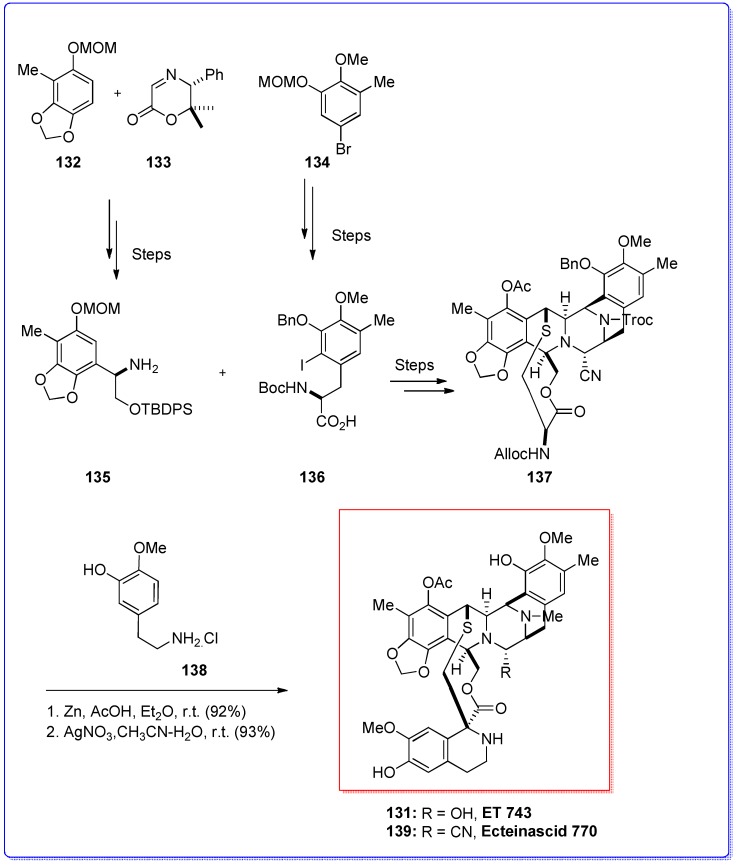
Total synthesis of Et 743 **131** and ecteinascidin 770 **139**.

Also, an asymmetric total synthesis of ecteinascidin 743 **131** was performed in 2002 by Fukuyama and co-workers [214]. In this approach, an Ugi four-component reaction, the intramolecular Heck reaction and PSR can be considered as main steps. By this pathway, total synthesis of Ecteinascidin 743 **131** was initiated from the reaction between two segments, amine **135** and carboxylic acid **136**. Significantly, an extremely functionalized (*R*)-phenyl glycinol derivative **135** was synthesized from the treatment of phenol **132** with iminolactone **133** (in several steps). Instead, (*S*)-iodophenyl alanine derivative **136** was provided from market purchasable 3-methylcatechol [215]. The treatment of amine **135** and carboxylic acid **136**, after several steps gave the corresponding ten-membered sulfide **137**. With the corresponding ten-membered sulfide **137** in hand, all that is necessary to complete the total synthesis of ecteinascidin 743 **131** is the production of the last tetrahydroisoquinoline scaffold. Removal of the Troc group followed *via* reductive alkylation reaction gave *N*-methylamine, whose Alloc group and allylether were instantaneously removed with Pd catalyst to afford the aminophenol. Based on the method reported by Corey [212], biomimetic trans amination reaction [216] gave the known α-ketolactone [217], and subsequent PSR with amine **138** provided ecteinascidin 770 **139** [218]. Lastly, construction of the labile hemiaminal from the aminonitrile affected by reaction with silver nitrate in acetonitrile–water to afford ecteinascidin 743 **131**, and afforded spectral data in complete agreement with those of the natural product (Scheme 26) [214].

Lemonomycin **145**, as a member of the tetrahydroisoquinoline group of antitumor antibiotics, which involves the quinocarcins, ecteinascidins and saframycins was initially extracted in 1964 from a fermentation broth of *Streptomyces candidus* and was known to have powerful antibiotic properties against *Staphylococcus aureus* and *Bacillus subtilis* [219]. The architecture of lemonomycin, though, was not clarified until 2000 [220], Besides the connectivity and relative stereochemistry of lemonomycin, the antibiotic property against methicillin-resistant *S. aureus* and vancomycin-resistant *Entero-coccus faecium*, as well as the cytotoxicity against a human colon tumor cell line were demonstrated. Lemonomycin is significant among the approximately 60 natural products and hundreds of synthetic equivalents in this group in which it contains a glycoside at C (18) [221].

**Scheme 27:**
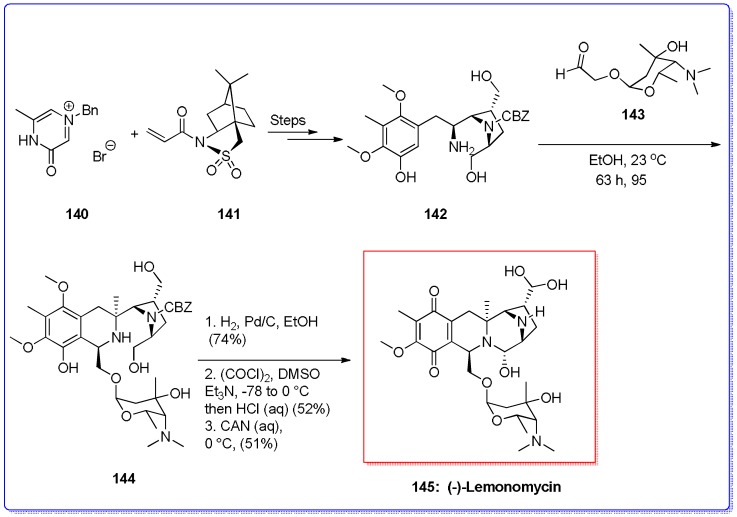
Total synthesis of Lemonomycine **145**.

Stoltz and co-workers in 2003 described the first total synthesis of the glycosylated tetrahydroisoquinoline antitumor antibiotic (−)-lemonomycin (15 steps from **140**). The merits of this convergent synthesis are the enantioselective dipolar cycloaddition which sets the stereochemistry of the glycone unit, a Suzuki coupling to link the diazabicycle to the aryl subunit, and a stereoselective PSR, which incorporates the aminoglycoside directly without the need for late-step glycosylation or masking group manipulations. This group demonstrated the first total synthesis of (−)-lemonomycin by usage of a stereoselective dipolar cycloaddition and a novel, diastereoselective PSR. For the total synthesis of (−)-lemonomycin, the reaction of bromide salt **140** and the Oppolzer sultam-derived acrylamide **141** [222] after several steps gave aminotriol **141**. Instead, an α-glycosyloxy acetaldehyde derivative **143** was synthesized from d-threonine [223,224]. In the following, the completion of the total synthesis now based on the success of the unprecedented PSR of the aminoglycosyloxy aldehyde **143** and the trifluoro acetic acid salt of amino triol **142** is given. Simple mixing of the two compounds in ethanol at ambient temperature provided the corresponding adduct **144** as a single diastereomer at C1 in 95% yield. Elaboration of tetrahydroisoquinoline **144** to the natural product was straight forward and included hydrogenolytic removal of the CBZ group, bis Swern oxidation, and reaction with CAN to give (−)-lemonomycin **145**. The completely synthetic precursor provided by this reaction was shown to be identical in all respects to a sample provided from natural sources (Scheme 27) [225].

Cribrostatin 4 **151** was extracted by Pettit and co-workers in 2000 in the Republic of Maldives from the blue sponge *Cribrochalina* collected [226]. Shortly afterwards, Kubo and co-workers [227] reassigned the architecture of reneiramycin H, extracted by Parameswaran and co-workers from *Haliclona cribicutis* [228], to be equal to that of cribrostatin 4 **151**. Cribrostatin 4 **151** belongs to a large group of complex tetrahydroisoquinoline natural products, that involves ecteinascidin 743 (Et 743), Et 597, and cyanosafracin [221].

**Scheme 28:**
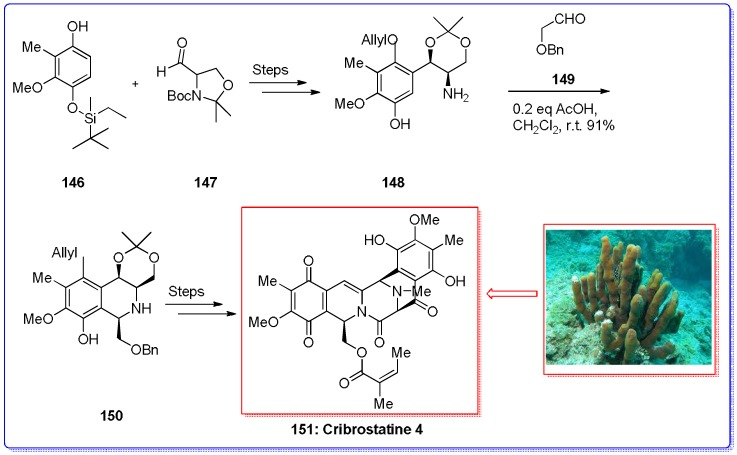
Total synthesis of Cribrostatine 4 **151**.

**Scheme 29:**
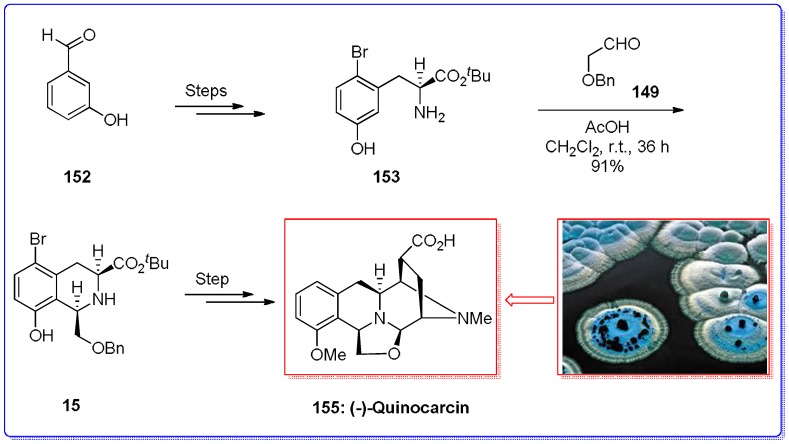
Total synthesis of Quinocarcin **155**.

A convergent total synthesis of cribrostatin 4 **151** was completed by Chen and co-workers in 2007 in a long linear sequence of 21 steps from the known phenol **146** in 4.3% overall yield (or in 26 steps from vanillin in 2.8% overall yield). Total synthesis of cribrostatin 4 **151** was initiated from the aldol condensation reaction of phenol **146 [229]** and the garneraldehyde **147** [230]. After several steps, the free aminophenol **148** was formed. The PSR of **148** and benzyloxyacetaldehyde **149** gave the 1,3-cistetrahydroisoquinoline **150** in 91% yield as a single diastereomer (*d.r*. >30:1) [137]. After several steps, total synthesis of cribrostatin 4 **151** was completed (Scheme 28) [231].

(−)-Quinocarcin **155**, a pentacyclic tetrahydroisoquinoline alkaloid [221], was extracted from the culture broth of *Streptomycesmeluno* V*inuceus* in 1983 by Takahashi and Tomita [232,233]. It showed significant antitumor properties against a number of tumor cell lines and its citrate salt (KW2152) has been used in clinic trials in Japan [234,235,236,237]. The anti-proliferative influence of (−)-quinocarcin was relatively explained by its ability to prevent RNA and/or DNA synthesis although, it was found that (−)-quinocarcin and (−)-tetrazomine showed cytotoxic properties [238]. The total synthesis of (−)-quinocarcin was obtained in a long linear sequence of 22 stages from 3-hydroxybenzaldehyde **152** in 16% overall yield. Remarkably, total synthesis of (−)-quinocarcin was initiated from 3-hydroxy benzaldehyde that after several steps gave the functionalized phenylalanine derivative **153**. The PSR of amino phenol **153** with benzoxy acetaldehyde **149** based on mild acidic tetrahydroisoquinoline conditions afforded **154** as a single diastereomer as a merely isolable stereomer in 91% yield. After several steps,(−)-quinocarcin **155** was synthesized in 16% overall yield (Scheme 29) [239].

Renieramycins, ecteinascidins and saframycins belong to marine bis tetrahydroisoquinoline alkaloids that are identified by their usual structural unit of five condensed six-membered rings including two tetrahydroisoquinoline scaffolds. These naturally occurring compounds show a series of significant biological activities for example antimicrobial and anti-tumor properties [240].

Renieramycins, involving jorunnamycins [241] and jorumycin [242], have become a large class in the marine bistetrahydroisoquinoline alkaloid group to date since they were first identified from the Mexican *bluesponge Renierasp*. by Frincke and Faulkner in 1982 [243]. Renieramycin-type alkaloids can be categorized into two sub-groups based on the C-21 functionalities. Some of these compounds contain carbinolamine or aminonitrile scaffolds at C-21 that are the required functional groups for linking to DNA and possibly other bio macromolecules in tumor cells [244]. Thus, a series of this subgroup of alkaloids for example **161** exhibited nanomolar inhibitory influences in a panel of human tumor celllines [241,242,245,246]. Although, these subgroup renieramycins, have an amide carbonyl residue at C-21 in place of carbinolamine and aminonitrile groups, they astonishingly retain their antitumor property [227,243,247]. For instance,(−)-renieramycin G **162**, a member in the second subgroup extracted from the Fijian sponge *Xestospongia caycedoi*, demonstrated cytotoxicity against human cancer cells [247].

**Scheme 30:**
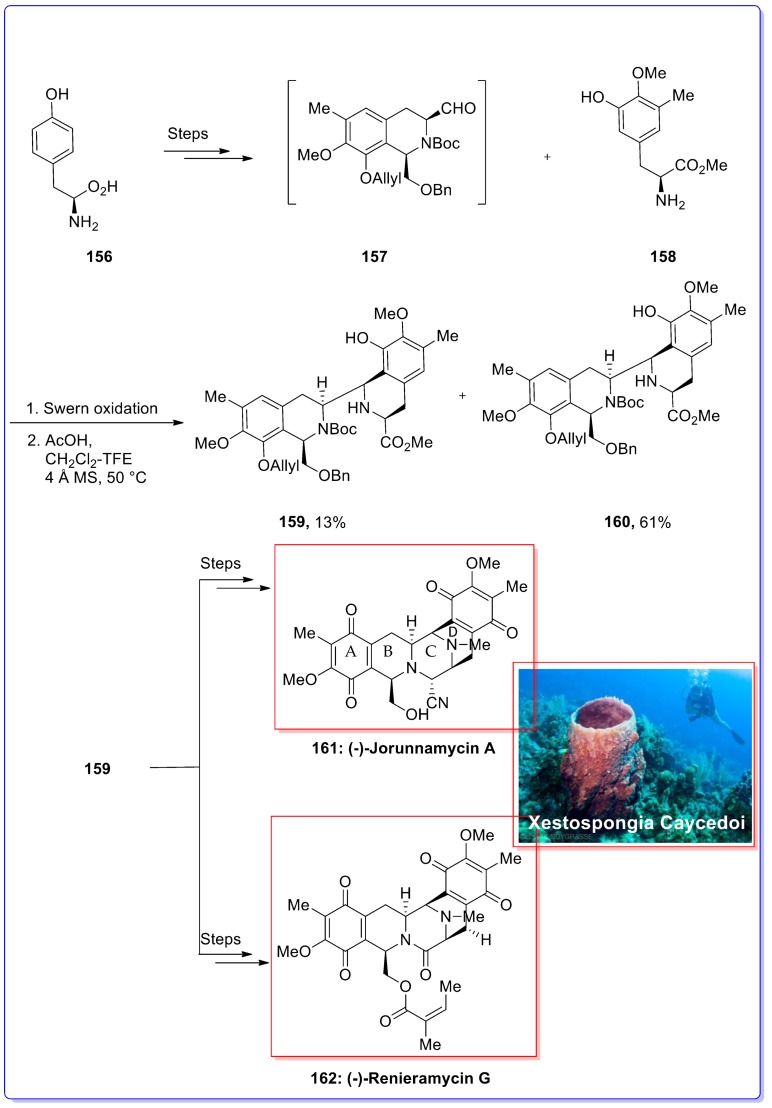
Total synthesis of renieramycin G **162** and jorunnamycin A **160** through PSR as the key step.

A flexible and useful method for the enantioselective synthesis of renieramycin-type antitumor alkaloids was demonstrated in 2014 by Chen and co-workers in that the stereoselective PSR of aldehyde **157** and aminoester **158** through regulating temperature and the automatic lactamization upon *N*-deprotection of the cyclization product were exploited to quickly construct the usual pentacyclic moiety. (–)-Renieramycin G and (−)-jorunnamycin A were obtaine in 19 steps from l-tyrosine with 15.8% and 14.3% overall yield respectively. The asymmetric total synthesis of (−)-renieramycin G and (−)-Jorunnamycin A was initiated from l-tyrosine **156**. After several steps, l-tyrosine **156** provided aldehyde **157** (the left partner) containing the A and B rings of the target. Instead, the trifunctionalized phenylalanine ester **158** (the right partner) was obtained from l-tyrosine with excellent overall yield [248]. With the two partners in hand, the step was set for creating the D ring through a PSR. Firstly, **157** and **158** were coupled. The reaction advanced rapidly based on this condition, and two cyclization isomers were obtained. The ratio of the less polar isomer **159** and the more polar isomer **160** was ca. 1:5. After several steps the natural products (−)-renieramycin G **162** and (−)-jorunnamycin A **161** were produced through various pathways. (−)-Jorunnamycin A **161** can be converted into other renieramycin alkaloids and their analogues [249,250,251,252,253,254] (Scheme 30) [255].

The 1-benzyltetrahydroisoquinoline architecture provides the basis for an enormous number of pharmaceuticals showing various mode of actions [256]. Tetrahydroprotoberberines (THPBs) include an additional methylene group to make a dibenzoquinolizidine ring system. A wide range of biological properties has been described for these alkaloids. To mention a few instances, C-8-unfunctionalized (−)-(*S*)-stepholidine exhibits a stimulating profile on the dopamine D1 and D2 receptors and has potential anti-nociceptive and antipsychotic properties [257]. Isocorypalmine has been established as an anti-cocaine therapeutic [258]. Tetrahydroprotoberberines having a group at the 8-position are less abundant in nature but were also demonstrated to show fascinating biological properties [259]. The regioisomers (+)-javaberine A **171** and B **172** include a third catechol-type aromatic ring and display a strong inhibitory influence on the lipopolysaccharide-induced tumor necrosis parameter [260]. The spiro alkaloid (−)-latifolian A **183** has an extra C-N bond, providing the quaternary nitrogen atom [261]. Latifolian A isolated from *Gnetum montanum* was demonstrated to exhibit anti-bacterial property against methicillin-resistant *Staphylococcus Aureus* [262]. Enantiopure 8-benzylprotoberberine derivatives were produced *via* two consecutive Pictet–Spengler condensations with masked 3,4-dihydroxyphenylacetaldehydes. The initial PSR to (+)-(*R*)-nor protosinome nine was normalized to 90% *ee* with 5 mol% of (*R*)-TRIP as chiral Brønsted acid (>99% *ee*). The second PSR did not need any catalyst, and its regioselectivity was powerfully dependent on the solvent: 99:1 para selectivity was provided in trifluoro-ethanol affording (+)-javaberine A; 81:19 *ortho* selectivity was achieved in polar aprotic solvents for the formation of (+)-javaberine B. Complete, natural diastereoselectivity was detected in the second PSR. Through selective catechol oxidation the spirocyclic alkaloid (−)-latifolian A was synthesized from masked (+)-javaberine A. Concise and extremely selective syntheses of the enantiopure target products have been performed. Starting from *N_p_*_s_-masked amine **163** overall yields of 48% for (+)-javaberine A, 35% for (+)-javaberine B and 41% for (−)-latifolian A have been obtained; *para* selectivity in the second PSR was increased to almost 100:0 in protic solvents, but, more significantly, the *ortho* selectivity was directed to 80:20 by a polar solvents, which opens a pathway to various bioactive 9-alkoxytetrahy-droprotoberberines.

Hiemstra and co-workers in 2016 demonstrated this synthetic method with the Pictet–Spengler condensation of **163** with masked dopal (dihydroxyphenylacetaldehyde **164**) [263]. An investigation to improve the reaction conditions and catalyst loading afforded the undesired observation in which lowering of the quantity of (*R*)-TRIP from 10 to 5 mol% afforded a slight increase of the enantioselectivity to a reproducible 90%. A probable clarification could be the fast construction of enamine **165** with concomitant release of H_2_O. Water links to the TRIP/iminium ion pair and has a negative effect on the *ee* [263]. Lower catalyst loading exhibits slow down the reaction and permits H_2_O to link to the drying agent Na_2_SO_4_ before the asymmetric cyclization occurs. The role of (*S*)-BINOL as a co-catalyst remains uncertain, but is considerable [263,264]. Upon selective cleavage of the Nps group from **166**, enantiopure (*R*)-**167** was extracted in satisfactory yield as an extremely insoluble compound by simple trituration. Elimination of the TBS group from **167** easily occurred to provide (+)-(*R*)-norprotosinomenine **168**, the precursor for the second Pictet–Spengler condensation. The free phenolic OH substituent in **168** was necessary for a smooth cyclization and made heating and strong acids unrequired. The aldehyde TBS-masked dopal **164** was again selected for its poor polarity and relative stability compared to dopal itself. From the first examinination, it became clear that the regioselectivity of this reaction was significantly identified by the solvent. Catalysts including (*R*)-or (*S*)-TRIP and thiourea catalysts slowed down this reaction and afforded incomplete reactions with a slight para preference. The effect of various solvents on the *ortho*/*para* regioselectivity of the Pictet–Spengler condensation of **164** and **168** was examined. The increasing H-bond donating character of the solvent matched the amount of *para*-functionalized product **170**, finishing with trifluoroethanol and hexafluoro-2-propanol as equally effective Addition of HOAc to dichloromethane as solvent had a slight influence on the product distribution (ca.1:1) and also reduced the reaction rate [265]. On a preparative scale the *para* selectivity was increased to 99:1 and the yield to 85%.

**Scheme 31:**
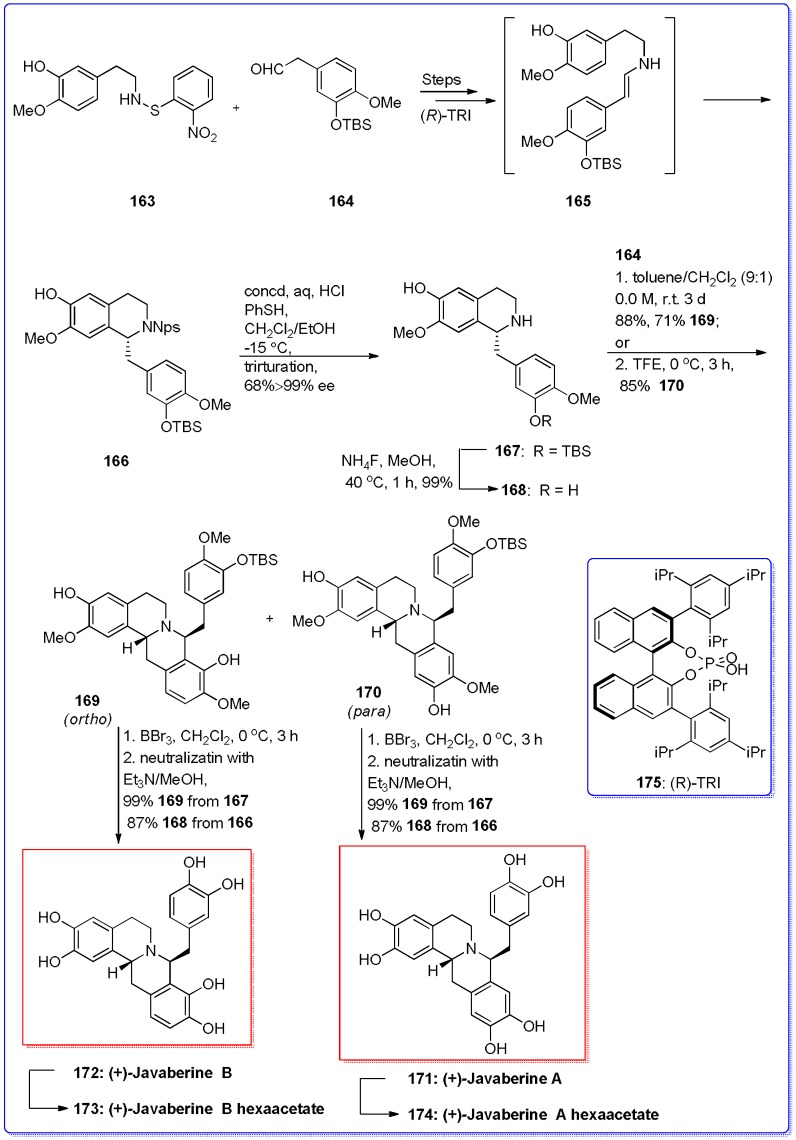
Total synthesis of javaberine A **171**, javaberine B **172,** javaberine B hexa-acetate **173** and javaberine A hexa-acetate **174** using PSR as key step.

To improve the construction of *ortho* product **169** aprotic, polar solvents were needed, and the solubility of the substrates was the merely restriction for more improvement. Lastly, an *ortho*/*para* ratio of 81:19 with 88% total yield was performed. In the last stages the OMe and OTBS groups were removed with borontribromide, providing the HBr salts of javaberine A **171** in 48% overall yield from **163** and of javaberine B **172** in 35% overall yield from **163**. Careful NMR analysis on these HBr salts, showed the formation of the desired free bases and the hexa-acetates **173** and **174**. The ^1^H and ^13^C-NMR spectra of **171**, **173**, and **174 [260]** and comparison of the sign of the optical rotations demonstrated the absolute configurations of the natural products as (8*R*,14*S*) (Scheme 31) [266].

**Scheme 32:**
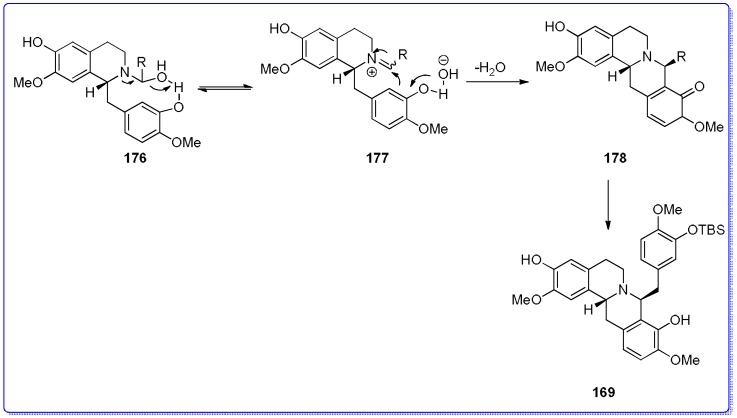
*ortho*-Selective Pictet–Spengler condensation.

In polar, aprotic solvents a transition state is exhibited in which the phenolic OH substituent protonates the originally provided aminal **176** in an intramolecular method to form the iminium salt **177**, as in all three natural product targets. Even trace quantities (<1%) of the undesired *cis* isomers were not detected (Scheme 32) [263].

**Scheme 33:**
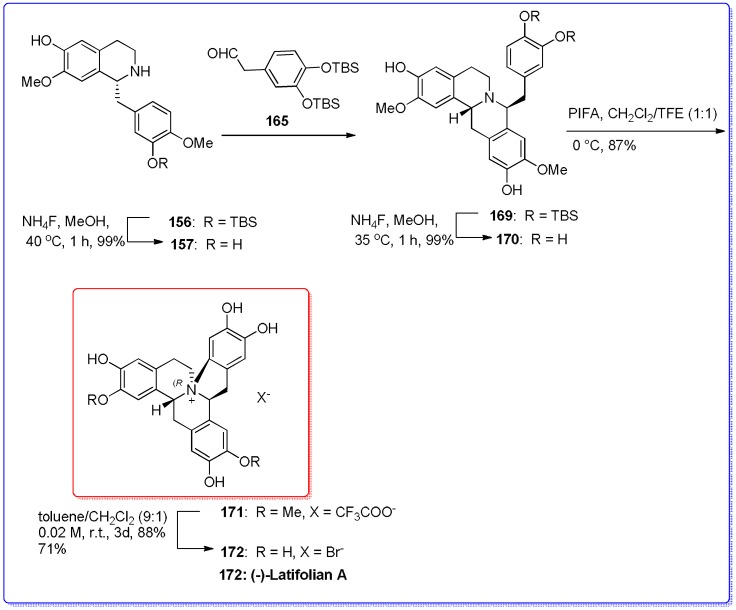
The synthesis of latifolian A **183**.

In addition, the formation of latifolian A **183** was initiated from (*R*)-norprotosinemonine **168** but needed a change of masking groups in the dihydroxyphenyl acetaldehyde PSR. Double-TBS-protected dopal **179** affords free catechol functionality upon desilylation, whereas the other two catechol groups stay masked as mono-methyl ethers. TFE as a regioselective Pictet–Spengler solvent again gave early entirely *para*-functionalized phenol **180** in excellent yield. Desilylation to **180** and oxidation with bis[(tri-fluoroacetoxy)iodo]benzene (PIFA) afforded the spirocyclic quaternary ammonium salt **182** as its bis-methyl ether, that was deprotected with HBr in HOAc to enantiopure latifolian A **183** (Scheme 33) [263].

## 3. Conclusions

In summary, PSR was discovered by Ame Pictet and Theodor Spengler, an important method for the synthesis of natural biologically active compounds. In this review, we aimed to underscore the significance and importance of PSR as an old reaction under a new perspective, its application in the important and new field of total synthesis of naturally occurring compounds. Nowadays, the PSR plays an important and key role in the total synthesis of natural products with diverse biological activities. Research results relating to the aforementioned points are growing fast in the literature and chemistry libraries. They reveal that the PSR is one of the most significant basic reaction categories in the total synthesis of the most important class of natural products known as alkaloids in nature. The important compounds from the biological point of view such as, ajmaline, vellosimine, talpinine, tryptophan, jamtine, talcarpine, alstonerine, jorumycin, renieramycin G, etc. have been synthesized *via* PSR as a determining step in their multistep total synthesis.
